# Pharmacokinetics of vitamin dosage forms: A complete overview

**DOI:** 10.1002/fsn3.3787

**Published:** 2023-11-09

**Authors:** Vrashabh V. Sugandhi, Rudra Pangeni, Lalitkumar K. Vora, Sagun Poudel, Sopan Nangare, Satveer Jagwani, Dnyandev Gadhave, Chaolong Qin, Anjali Pandya, Purav Shah, Kiran Jadhav, Hitendra S. Mahajan, Vandana Patravale

**Affiliations:** ^1^ College of Pharmacy & Health Sciences St. John's University New York New York USA; ^2^ Department of Pharmaceutics Virginia Commonwealth University Richmond Virginia USA; ^3^ School of Pharmacy Queen's University Belfast Belfast UK; ^4^ Department of Pharmaceutics H. R. Patel Institute of Pharmaceutical Education and Research Shirpur Maharashtra India; ^5^ KLE College of Pharmacy KLE Academy of Higher Education and Research Belagavi Karnataka India; ^6^ Department of Pharmaceutics Sinhgad Technical Education Society Sinhgad Institute of Pharmacy Pune Maharashtra India; ^7^ Department of Pharmaceutical Sciences and Technology Institute of Chemical Technology Mumbai India; ^8^ Thoroughbred Remedies Manufacturing TRM Industrial Estate Newbridge Ireland; ^9^ Department of Pharmaceutics R. C. Patel Institute of Pharmaceutical Education and Research Shirpur Maharashtra India

**Keywords:** drug delivery, nanocarriers, pharmaceutical dosage form, pharmacokinetics of vitamins, vitamins

## Abstract

Vitamins are crucial for sustaining life because they play an essential role in numerous physiological processes. Vitamin deficiencies can lead to a wide range of severe health issues. In this context, there is a need to administer vitamin supplements through appropriate routes, such as the oral route, to ensure effective treatment. Therefore, understanding the pharmacokinetics of vitamins provides critical insights into absorption, distribution, and metabolism, all of which are essential for achieving the desired pharmacological response. In this review paper, we present information on vitamin deficiencies and emphasize the significance of understanding vitamin pharmacokinetics for improved clinical research. The pharmacokinetics of several vitamins face various challenges, and thus, this work briefly outlines the current issues and their potential solutions. We also discuss the feasibility of enhanced nanocarrier‐based pharmaceutical formulations for delivering vitamins. Recent studies have shown a preference for nanoformulations, which can address major limitations such as stability, solubility, absorption, and toxicity. Ultimately, the pharmacokinetics of pharmaceutical dosage forms containing vitamins can impede the treatment of diseases and disorders related to vitamin deficiency.

## Graphical abstract

This review highlights the importance of the pharmacokinetics of vitamins in maintaining human health and offers reliable evidence for their efficacy in the human body. Effective delivery of vitamins is desired to avoid their deficiency, and therefore, alternate routes of administration could improve the pharmacokinetics of vitamins. Nanoformulation‐based delivery is a feasible strategy to improve vitamin delivery.

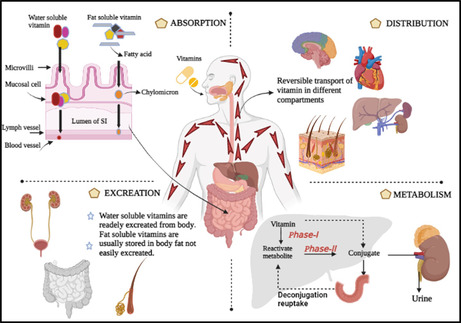

## 1 INTRODUCTION

Vitamins are a specific group of organic compounds essential for bodily metabolic processes, as they cannot be produced in sufficient quantities by the body and must be obtained from the diet at trace levels (<1 g/day) (Borel & Desmarchelier, 2018; Combs & McClung, 2016). Other necessary nutrients, such as dietary minerals, essential fatty acids, and essential amino acids, are not included in the term “vitamin,” nor does it encompass a wide variety of other nutrients that support health but are consumed less frequently. Today, the common term “vitamin” is part of everyday language, emphasizing the connection between diet and health. Vitamin deficiencies remain a global concern. They often go clinically unnoticed unless they reach severe levels, but even mild deficiencies can have significant consequences. Vitamin deficiencies can affect individuals of all ages, but they are particularly common in pregnant and breastfeeding women, as well as young children, who have increased requirements for these nutrients and are more susceptible to their absence. These consequences encompass outcomes such as increased susceptibility to infectious diseases, anemia, maternal mortality, impaired cognitive development, and hindered physical growth (Griffiths, 2020).

The World Health Organization (WHO) estimates that over two billion people worldwide suffer from micronutrient deficiencies. Among them, approximately 149 million children under the age of 5 are estimated to be stunted, 45 million are estimated to be wasted, and 38.9 million are overweight or obese. These conditions may be attributed, in part, to a lack of essential vitamins such as vitamin A, vitamin D, various B vitamins, and essential minerals (Darnton‐Hill, 2019; Keats et al., 2021). Vitamin inadequacies have a much greater impact on women's health and reproductive outcomes than was previously understood (Darnton‐Hill, 2019). As a result, vitamins have been recognized as essential nutrients that must be acquired through the diet to maintain good health and treat deficiencies. Dietary Reference Intakes (DRIs) provide recommended guidelines for the dietary intake of micronutrients. These standards were established in 1997 by the Food and Nutrition Board of the Institute of Medicine. The committee panel endorses recommendations regarding food intake, nutrition, and the quantitative requirements of vitamins. The reports generated by the DRIs are intentionally designed with a focus on ensuring the safety and quality of nutrients (Institute of Medicine, 2011).

The pivotal role of vitamins is to function as coenzymes, facilitating the transformation of apoenzymes into holoenzymes, thereby regulating both catabolic and anabolic reactions. Several water‐soluble vitamins serve as coenzyme (Freeland‐Graves & Bavik, 2003). The categorization of vitamins based on their solubility is a fundamental concept in nutrition and biochemistry. Vitamins are essential organic compounds that the human body requires in small quantities for various metabolic and physiological processes. Their solubility plays a crucial role in how they are absorbed, transported, and utilized by the body. Table 1 summarizes the brief summary of vitamins. In short, there are two primary categories of vitamins based on solubility: water‐soluble vitamins and fat‐soluble vitamins. Water‐soluble vitamins: These vitamins dissolve in water and are not stored to a significant extent in the body. Instead, they are readily absorbed in the small intestine and are transported through the bloodstream. Any excess water‐soluble vitamins that the body does not immediately use are typically excreted in the urine. The water‐soluble vitamins include vitamin B1 (thiamine), B2 (riboflavin), B3 (niacin), B5 (pantothenic acid), B6 (pyridoxine), B8 (biotin), B9 (folic acid), B12 (cobalamin), and vitamin C (ascorbic acid). These vitamins play vital roles in processes like energy metabolism, cell growth, and immune function. Fat‐soluble vitamins, on the other hand, do not dissolve in water but are soluble in fats and oils. Unlike water‐soluble vitamins, fat‐soluble vitamins are stored in the body's fatty tissues and liver. This storage allows the body to draw upon these reserves when dietary intake is insufficient. The fat‐soluble vitamins include vitamin A (retinol), vitamin D (calciferol), vitamin E (tocopherol), and vitamin K (phylloquinone). These vitamins are essential for various functions, such as vision, bone health, antioxidant defense, and blood clotting. Understanding the solubility of vitamins is crucial because it impacts their absorption, transportation, and storage within the body. While both types of vitamins are essential for health, it is important to maintain a balanced diet that provides an adequate supply of both water‐soluble and fat‐soluble vitamins to support overall well‐being and prevent deficiencies (Fitzpatrick et al., 2012). Furthermore, the classification of vitamins is based on their biological and chemical activity. Consequently, the term “vitamin” may encompass various vitamin compounds, all of which exhibit biological activity associated with a specific vitamin. For instance, “vitamin A” encompasses compounds such as retinal, retinol, and numerous carotenoids. It is important to note that vitamins can undergo interconversion within the human body (Bhagavan, 2002; Combs & McClung, 2016). Fat‐soluble vitamins are typically absorbed into the lacteals of the small intestine via chylomicrons with the assistance of bile salts. They are then transported through the lymphatic system and eventually released into the bloodstream (Karunaratne et al., 2017). Fat‐soluble vitamins are often stored in the liver or fatty tissues of our bodies until needed, which means they do not typically require frequent ingestion (Coulston et al., 2001). The absorption, transport, activation, and utilization of these fat‐soluble vitamins involve enzymes or other proteins whose synthesis is genetically controlled (Bhagavan & Ha, 2015). A deficiency in one of these proteins can result in a disease that closely resembles the effects of a dietary deficiency (as shown in Table 1). Vitamin A, especially in the form of carotenoids such as α‐ and β‐carotene, and β‐cryptoxanthin, can be enzymatically converted into retinol in the human body. This conversion is facilitated by the enzyme β‐carotene oxygenase 1 (BCO1), and as a result, these compounds are referred to as pro‐vitamin A (proVA) carotenoids. Vitamin D exists in two primary forms: vitamin D2 (ergocalciferol) and vitamin D3 (cholecalciferol), both of which are essential for various physiological functions (see structures in Table 1) (Borel & Desmarchelier, 2018). In humans, the synthesis of vitamin D through exposure to sunlight is highly variable and depends on numerous factors, including the duration of sun exposure, season, clothing, time of day, latitude, atmospheric conditions, sunscreen use, and skin pigmentation. However, a significant proportion of people rely on dietary vitamin D from food supplements to meet their specific needs (Hill et al., 2012). Similar to vitamin A, vitamin D is a pro‐hormone that becomes active after undergoing two hydroxylations in the liver (Borel & Desmarchelier, 2018). Vitamin D plays a critical role in maintaining bone health and regulating blood calcium levels. Additionally, it is involved in various other biological functions, including immune response, cell apoptosis, and cell proliferation.

**TABLE 1:** Vitamins: Their vitamers, structure, function, and deficiency symptoms.

Vitamin	Vitamers	Structure	Physiological function	Deficiency symptoms	Ref.
A	Retinol Retinal Retinoic acid	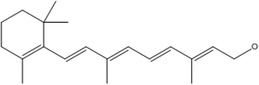	Visual pigments, epithelial cell differential, and immune responses	Xerophthalmia, delayed growth, dry skin, infertility and trouble conceiving	Combs and McClung (2016); Fitzpatrick et al. (2012); Shenkin (2008); Ramakrishnan et al. (2004); Ko et al. (2014)
D	Cholecalciferol (D3)	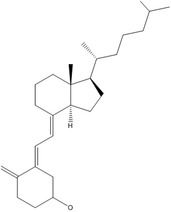	Calcium homeostasis, bone metabolism, and transcription factor	Joint pain, muscle weakness, and frequent infections	DeLuca (2004); Wang et al. (2017)
E	α‐Tocopherol	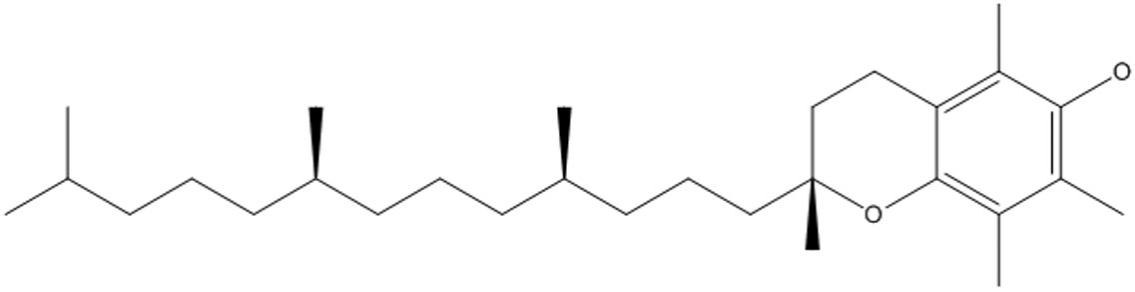	Membrane antioxidant, and cellular signaling	Neuromuscular abnormalities	Brigelius‐Flohé and Traber (1999)
K	Phylloquinone (K1)	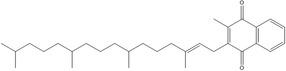	Blood clotting, and calcium metabolism	Hemorrhaging, osteopenia, and/or osteoporosis	Mladěnka et al. (2022)
C	Ascorbic acid Dehydroascorbic acid	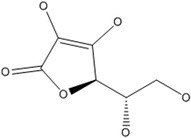	Collagen stabilization and maturation, synthesis of hormones (noradrenaline/adrenaline and peptide hormones), synthesis of carnitine, gene transcription, and regulation of translation via different mechanisms	Scurvy, and dyspnea	Doseděl et al. (2021)
B1	Thiamin	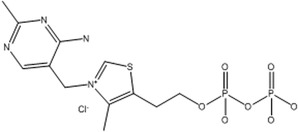	Coenzyme for decarboxylations of 2‐keto acids (e.g., pyruvate) and transketolations	Anorexia and weight loss, mental changes, and muscle weakness	Combs and McClung (2016); Shenkin (2008)
B2	Riboflavin	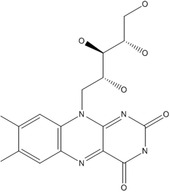	Coenzyme in redox reactions of fatty acids and the tricarboxylic acid (TCA cycles)	Cheilosis, angular stomatitis and glossitis and a seborrheic dermatitis	Combs and McClung (2016); Shenkin (2008)
Niacin (B3)	Nicotinic acid	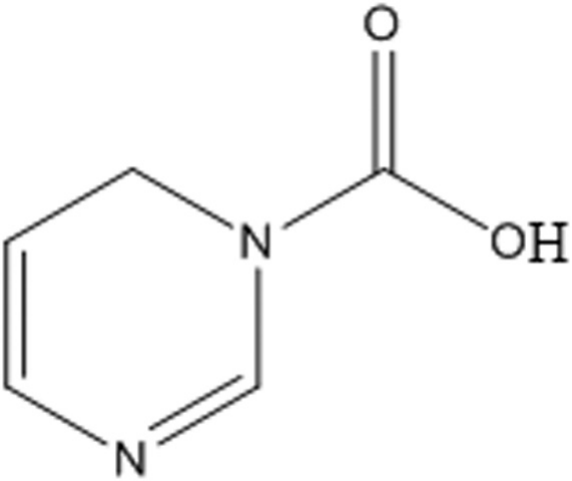	Coenzyme for several dehydrogenases, special importance in both carbohydrate and fat metabolism	Pellagra, depression, and memory loss	Combs and McClung (2016); Shenkin (2008)
B5	Pantothenic acid	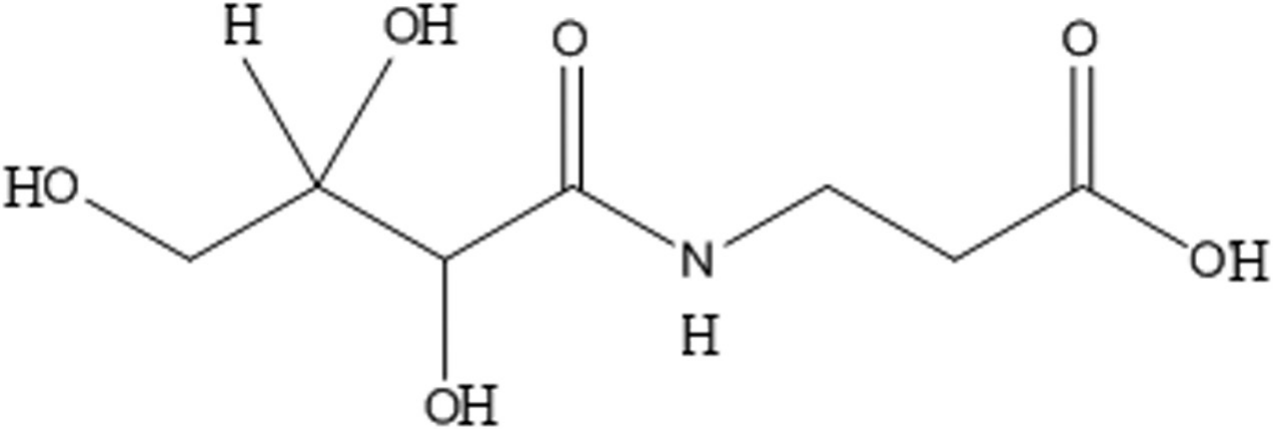	Coenzyme in fatty acid metabolism	Muscle cramps, numbness or burning sensation in hands or feet, and restlessness	Shenkin (2008)
B6	Pyridoxine	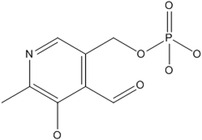	Coenzyme in amino acid metabolism	Anemia (children), premenstrual symptoms, lesions of lips and skin	Combs and McClung (2016); Shenkin (2008)
B7	Biotin	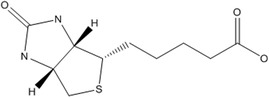	Coenzyme for carboxylations. Involves gluconeogenesis, biosynthesis of fatty acids, and catabolism of certain amino acids and fatty acids.	Neurological disorder, growth retardation, and dermal abnormalities	Said (2004a)
B9	Folic acid	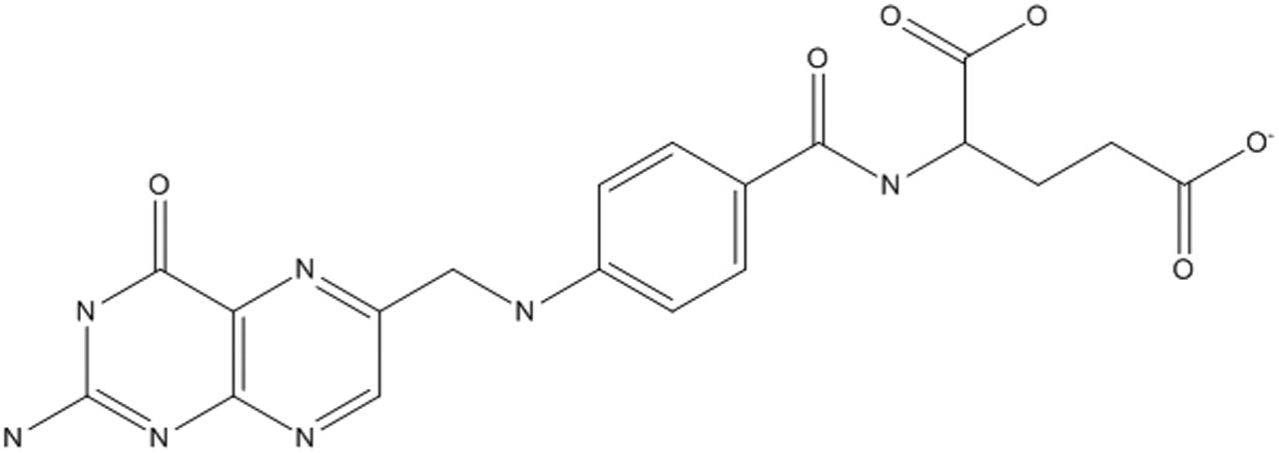	Coenzyme in single–carbon metabolism (synthesis of DNA and RNA, and metabolism of several amino acids)	Megaloblastic anemia, and growth retardation	Said (2004a)
B12	Cobalamin	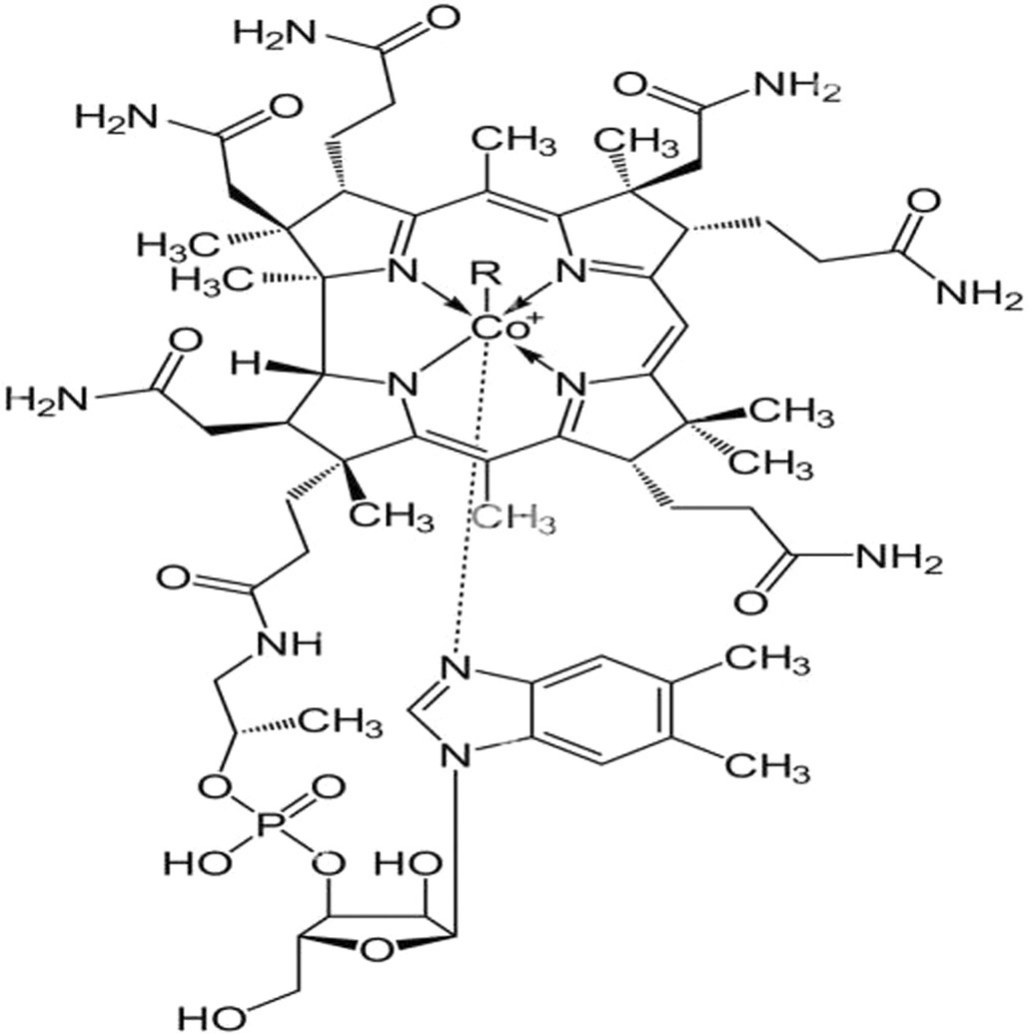	Coenzyme in the metabolism of propionate, amino acids, and single carbon units (DNA synthesis, metabolism of amino acids, and fatty acid synthesis)	Neurological disorder (nerve damage, tingling sensation, etc.), and anemia.	Halsted (2003)

The fat‐soluble antioxidant metabolite known as “vitamin E” is a crucial dietary component that includes tocopherol and tocotrienols. Each of these naturally occurring compounds consists of four isomers (α, β, γ, and δ) determined by the position and number of methyl groups on the chromanol ring. While a diet rich in γ‐tocopherol serves as the primary source of vitamin E, α‐tocopherol predominates in the bloodstream and is associated with various biological activities in humans. Vitamin E undergoes metabolism in the liver, and only α‐tocopherol is resecreted into the bloodstream, a process facilitated by the hepatic α‐tocopherol transfer protein (α‐TTP). This protein plays a critical role in maintaining a stable level of α‐tocopherol in the blood, which is essential because defects in the α‐TTP gene can result in vitamin E deficiency (Eggermont, 2006). Another fat‐soluble vitamin, vitamin K, acts as a coenzyme in the post‐translational carboxylation of glutamate residues in multiple proteins, leading to the activation of various proteins, including coagulation factors such as VII, IX, X, and prothrombin (Shearer et al., 2012).

Water‐soluble vitamins constitute a diverse group of organic compounds that share the common characteristic of being essential for normal cellular functions. In contrast to fat‐soluble vitamins, water‐soluble vitamins are not stored in the body and must be regularly included in the diet to prevent deficiency. There are nine water‐soluble vitamins: the B vitamins (folate, thiamine, riboflavin, niacin, pantothenic acid, biotin, vitamin B6, vitamin B12) and vitamin C. A deficiency in any of these water‐soluble vitamins can lead to clinical syndromes with the potential for severe morbidity and mortality (Lykstad & Sharma, 2021). These vitamins are readily absorbed by enterocytes in the small intestine through membrane transport processes. The absorption of vitamin B12, also known as cobalamin (Cbl), involves multiple processes, including the use of at least four different binding proteins for its journey from the stomach to the ileum (Halsted, 2003). Hence, the gastrointestinal system plays a critical role in preserving and regulating the body's micronutrient homeostasis. Reduced absorption of these compounds within the intestines can result in vitamin deficiency (Said, 2004a). Furthermore, water‐soluble vitamins serve as essential enzyme cofactors in a wide range of metabolic reactions. Riboflavin, niacin, and vitamin C are pivotal in redox reactions, while thiamine and biotin play roles in macronutrient metabolism. Folate, vitamin B12, pyridoxine, and riboflavin are significant contributors to the regulation of S‐adenosylmethionine production and DNA synthesis (Halsted, 2003). This review aims to provide an overview of the current understanding of vitamin pharmacokinetics, including factors influencing their biodistribution, and emphasizes the importance of pharmacokinetic analysis for more robust clinical studies. It is worth noting that limited information is currently available regarding the pharmacokinetics of these various vitamins. We hope that this will encourage further research into the pharmacokinetics of vitamins in both health and disease contexts.

## 2 THE IMPORTANCE OF VITAMIN PHARMACOKINETICS ANALYSIS

Whether administered orally or parenterally, vitamins enter the bloodstream, distribute to peripheral regions, and equilibrate with various organs (such as the liver and kidneys), tissues (including muscle and adipose tissue), and cells (including red blood cells) (Alpers, 2011). The degree of affinity between vitamins and these internal compartments affects the speed of redistribution and, consequently, the length of the vitamin's terminal half‐life. While a two‐compartment model applies to some vitamins, a multicompartmental distribution better fits the characteristics of most vitamins, such as vitamin A and vitamin C. The concentration of these vitamins in blood plasma determines their clearance from the bloodstream as they pass through metabolically active organs like the liver and kidneys. Metabolites generated in the liver follow the biliary route for active vitamin forms, such as 25‐hydroxyvitamin D [25(OH)D], retinoic acid, cobalamin, and N‐5, methyl tetrahydrofolate, following enterohepatic reabsorption. Some vitamins are metabolized into active forms in the hepatic bloodstream (e.g., folate to tetrahydrofolate), while others are stored in an unmetabolized state, like retinol palmitate in the liver, white blood cells (e.g., vitamin C and folate), and adipose tissues (e.g., vitamins A, D, and E). The movement of vitamins between plasma and tissue cells depends on their nonprotein‐bound, nonionized form and free tissue concentration. The concentration within the cell is influenced by factors such as the molecule's affinity with plasma and cell surface proteins, cellular permeability factors, and the effectiveness of generating stores like active protein binding sites. Due to these intricate processes in absorption, distribution, metabolism, and excretion, and their interdependence, plasma concentration alone cannot precisely predict the cellular efficacy in achieving a concentration of the vitamins or their active metabolites. Below, we provide a few examples of vitamins that underscore the significance of considering pharmacokinetic factors in understanding their bioavailability and therapeutic effectiveness.

### 2.1 Transportation pathway for vitamin C

**FIGURE 1:**
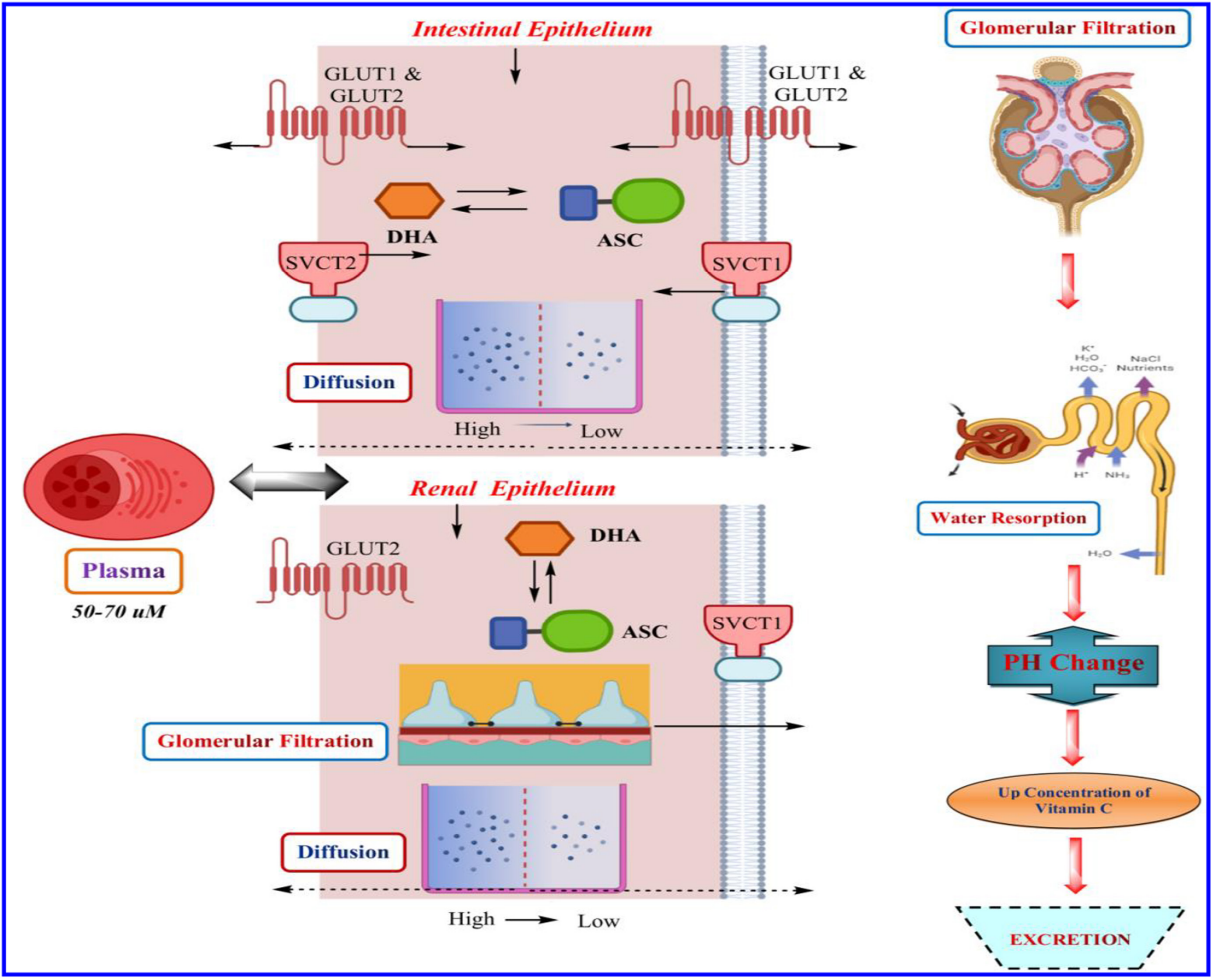
The diagram elucidates the ADME mechanism of vitamin C.

Water‐soluble vitamins serve as essential enzyme cofactors in a wide ranVitamin C exhibits dose‐dependent pharmacokinetics, primarily relying on saturable sodium‐dependent vitamin C transporters (SVCTs) (see Figure 1). Its homeostasis in the body depends on the tissue‐specific expression of SVCTs, resulting in concentrations of vitamin C as high as 10 mM in the brain and adrenal glands, and a minimum of approximately 0.2 mM in the muscles and cardiac region. The transition from zero‐ to first‐order pharmacokinetics introduces complexity in accurately calculating and understanding its dose‐dependent pharmacological effects, often leading to erroneous conclusions (Lykkesfeldt & Tveden‐Nyborg, 2019).

Administered vitamin C is primarily absorbed in the intestinal epithelium via membrane transporters in the apical brush border membrane. This absorption can occur in two ways: as dehydroascorbic acid (DHA) through facilitated diffusion facilitated by the glucose transporters GLUT2 or GLUT3, or as the activated form via sodium‐dependent vitamin C transporter (SVCT) 1. Within the cell, DHA is efficiently converted to ascorbate (ASC) or transported to the circulation with the assistance of GLUT1 and GLUT2 in the basolateral membrane. This process maintains a low intracellular concentration of ASC while promoting further DHA absorption. ASC is then transported to the plasma, possibly with the aid of enhanced diffusion through volume‐sensitive anion channels. In addition to the intestinal epithelium, ASC may also be reabsorbed from plasma by SVCT2, found in the basolateral membrane of renal tubule cells. The kidneys eliminate ASC through glomerular filtration into the renal tubule lumen. While diffusion from the luminal surface could potentially contribute to overall uptake, reabsorption is primarily attributed to SVCT1 transporters in the apical membrane. Although not confirmed in vivo, it is believed that DHA may also be reabsorbed in renal tubule cells. However, due to the extremely low plasma levels of DHA, its contribution to reabsorption is considered insignificant. Both ASC and DHA can enter the bloodstream through passive and facilitated diffusion, similar to their uptake in the intestinal epithelium. DHA can be transported to the plasma via GLUT2 transporters present in the basolateral membrane.

### 2.2 Regulation of vitamin D

**FIGURE 2:**
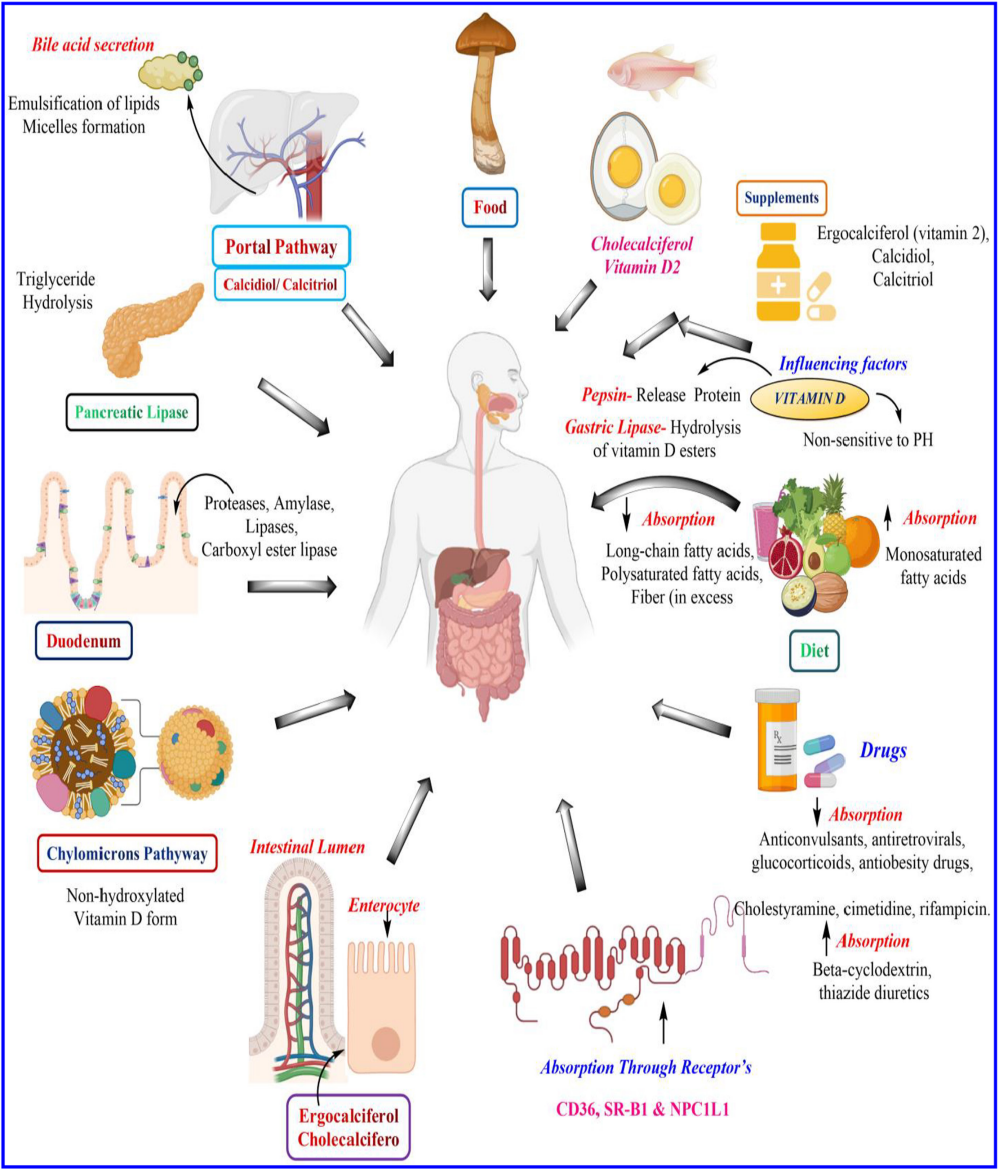
Absorption enzymatic transformation and excretion of vitamin D.

While vitamin D is typically taken in daily doses, it is now available in weekly, biweekly, and monthly boluses. The rationale behind these alternative dosing schedules is to achieve rapid absorption of fat‐soluble cholecalciferol by adipose tissue, ensuring long‐term availability (see Figure 2). In a randomized clinical trial involving vitamin D‐deficient patients, the pharmacokinetic and safety parameters were examined at total cumulative doses of 588,000 IU, 600,000 IU, and 600,000 IU in three groups (administered daily, weekly, and biweekly). The study found that normal serum levels of 25(OH)D, a vitamin D metabolite, were achieved in all groups, regardless of the dosing regimen. Interestingly, the results indicated that 25(OH)D exposure (at equal cumulative dosing) was higher with daily dosing compared to weekly dosing, which, in turn, was slightly superior to the biweekly schedule. This variation in exposure was attributed to the activity of enzymes, particularly the highly efficient/low‐saturated 25‐hydroxylase or the lesser‐induced catabolizing 24‐hydroxylase, which played a role in controlling the pharmacokinetics (Fassio et al., 2020).

From the diagram, we can see that natural sources of vitamin D are eggs, fish, and monosaturated fatty acid‐containing food. Vitamin D is obtained from external sources such as supplements in the form of cholecalciferol, ergocalciferol, calcitriol, and calcidiol. Pepsin can help separate vitamin D from proteins, and gastric lipase may partially hydrolyze vitamin D esters so that they can be absorbed during vitamin D digestion in the stomach. Amylases, protease, and lipase enzymes in the duodenum liberate vitamin D from the food matrix for intestinal absorption. The absorption of cholecalciferol and ergocalciferol by enterocytes is mediated by intestinal cell membrane receptors such as SR‐BI, CD36, and NPC1L1. Vitamin D is packed into chylomicrons and delivered to the bloodstream via the lymphatic system through the chylomicrons route. In the portal pathway, vitamin D travels from the colon to the liver through the portal system, where it is circulated with amino acids and carbohydrates. Diet: A long‐chain fatty acid diet contains a high number of polyunsaturated fatty acids, and an abundance of dietary fiber may reduce the absorption and bioavailability of vitamin D. In contrast, a diet containing high monounsaturated fatty acids may boost absorption and bioavailability. Medicines: Several drugs may inhibit the absorption of vitamin D. Scavenger receptor class B type 1 (SR‐BI), for Cluster Determinant 36 (CD36), and Niemann‐Pick C1‐Like 1 (NPC1L1).

### 2.3 Distribution and excretion mechanism of vitamin E

When vitamin E is administered orally, it leads to the formation of chylomicrons, which are triglyceride lipoprotein derivatives containing fatty acids and cholesterol. These chylomicrons are produced in the intestines and follow a lymphatic pathway to reach various tissues and organs, including the liver. While circulating in the bloodstream, lipase enzymes facilitate the hydrolysis of chylomicrons, resulting in the release and distribution of vitamin E to different tissues. The remnants of both vitamin E and chylomicrons are metabolized by CYPP450 enzymes. Following metabolism, they are excreted via bile, urine, and feces (Mohd Zaffarin et al., 2020).

Results from clinical studies comparing the pharmacokinetic parameters of α‐tocopherol and tocotrienol following oral supplementation indicate that α‐tocopherol has a T_max_ (time to maximum concentration) of approximately 6 h post‐meal, whereas tocotrienols exhibit a T_max_ of 3 to 4 h. Additionally, these findings reveal that α‐tocopherol reaches a higher peak plasma concentration (C_max_) compared to tocotrienols, with a half‐life (t_1/2_) that is approximately 4.5‐ to 8.7‐fold longer for α‐tocopherol than for two different tocotrienol isomers. Due to the presence of multiple rate‐limiting pharmacokinetic parameters and their respective fates, vitamin E, particularly tocotrienols, is considered a candidate for poor oral bioavailability. This necessitates a twice‐daily dosing frequency to maintain adequate plasma concentration and therapeutic effectiveness (Mahipal et al., 2016; Qureshi et al., 2015, 2016). As expected, subcutaneous parenteral administration of tocotrienol resulted in a Tmax of 1 h, in contrast to oral administration (Satyamitra et al., 2012). In vivo studies in rats also showed that intramuscular and intraperitoneal injections resulted in lower plasma concentrations compared to oral administration due to the absence of absorption‐enhancing micelle formation (Mahipal et al., 2016).

## 3 EFFECT OF OBESITY ON THE REGULAR FUNCTION OF VITAMINS

### 3.1 Role of vitamins in obesity regulation

Vitamin A levels in the organs of obese individuals are significantly reduced. Even with diets providing sufficient vitamin A, obese mice, whether induced by a high‐fat diet or genetic mutations, exhibit significantly lower levels of retinol in various organs, including the liver, lungs, pancreas, and kidneys (Trasino et al., 2015). Obesity leads to impaired vitamin A transcriptional signaling, resulting in reduced levels of retinoic acid receptor (RARα, RARβ2, RARγ) mRNAs and retinol‐binding proteins (RBP1) in vitamin A‐storing stellate cells. Additionally, obese individuals experience increased systemic and adipose tissue‐specific oxidative stress (Keaney Jr. et al., 2003). Upregulation of the renin‐angiotensin system and a decrease in erythrocyte glutathione and glutathione peroxidase antioxidant defense mechanisms are common mechanisms that contribute to increased oxidative stress in obese individuals (Barton et al., 2000; Manna & Jain, 2015). This heightened oxidative stress results in increased oxidative catabolism of lipid‐soluble nutrients like vitamin E and β‐carotene, which play a protective role in preventing the oxidation of LDL (Jialal & Fuller, 1995; Kimmons et al., 2006).

### 3.2 Function of vitamin C in obesity management

Numerous studies have consistently demonstrated an inverse relationship between body weight and vitamin C status, particularly in obese individuals who are often critically deficient in vitamin C (Pearson et al., 2017; Schleicher et al., 2009). The low vitamin C levels in obese individuals can be attributed, in part, to increased oxidative stress, leading to higher catabolism of vitamin C (Carr et al., 2017). Folic acid deficiency is also reported among obese individuals, and this deficiency can be attributed to several potential factors. Common reasons for low vitamin C levels in obese individuals include reduced dietary intake and absorption, increased catabolism of vitamin C, sequestration in adipose tissue, imbalanced gut microbiota profiles affecting vitamin B12 metabolism, adipocyte dysfunction, and reduced protein synthesis, among others (Castaner et al., 2018; Krishnaveni et al., 2009; Sun et al., 2019).

### 3.3 Role of vitamin D in obesity management

Obesity is a widespread global health concern driven by the accessibility of low‐cost, high‐calorie, and low‐nutrition foods. It often results in malnutrition, leading to deficiencies in various micronutrients, vitamins, minerals, and more (Astrup & Bugel, 2019; Kobylinska et al., 2022). Numerous studies have revealed a high prevalence of deficiency and insufficiency in both water‐soluble vitamins (B and C) and fat‐soluble vitamins (D3 and E) among obese individuals (Nath et al., 2017; Poitou Bernert et al., 2007). Aasheim et al. reported significant differences in the levels of six out of nine vitamins between obese patients with a BMI above 35–40 and healthy controls. Both male and female obese patients exhibited significantly lower mean serum concentrations of vitamins A, B6, C, D, and E. However, vitamins A, B1, B2, and B12 showed no significant difference between the two groups (Aasheim et al., 2008). One of the notable deficiencies seen in obese individuals is in 25‐hydroxyvitamin D (25 (OH)D), a clinical indicator of vitamin D status. Vitamin D tends to be sequestered by adipose tissues and diluted within the body's fat mass, leading to lower levels of circulating 25‐hydroxyvitamin D concentrations in obese individuals. Although vitamin D is taken up and stored in the adipose tissues of obese patients, it may not be released when needed (Earthman et al., 2012; Wortsman et al., 2000). Supporting this observation, a study by Blum et al. found an inverse relationship between the amount of fat tissue and serum vitamin D3 concentration when measuring adipose tissue vitamin D3 concentrations in 17 obese subjects (Blum et al., 2008).

## 4 EFFECT OF DRUG INTERACTION ON THE PHARMACOKINETICS OF VITAMINS THROUGH GIT

Nutrient–drug interactions can significantly impact the digestion, absorption, metabolism, catabolism, and excretion of nutrients. These interactions may arise due to various factors, including physical, chemical, physiological, or pathophysiological relationships between nutrients and drugs (Chan, 2013). Water‐soluble vitamins B and C have been studied for their potential interactions with drugs. For instance, the prolonged use of proton pump inhibitors (PPIs) for at least 12 months has been associated with an increased incidence of vitamin B12 deficiency, particularly in individuals over the age of 65. Omeprazole, for example, hinders the absorption of protein‐bound vitamin B12 in the intestine (Schenk et al., 1996; Valuck & Ruscin, 2004). Enzymes such as pepsin play a crucial role in releasing dietary vitamin B12 from dietary proteins, making it available for absorption. This process requires stomach acid to activate pepsin from its pepsinogen precursor. Without adequate stomach acid, vitamin B12 cannot be separated from food protein, and it cannot bind to R‐proteins, preventing pancreatic digestion (Heidelbaugh, 2013).

### 4.1 Proton pump inhibitor therapy (PPIT) on the upregulation of vitamin C

The use of proton pump inhibitors (PPIs) has also been linked to reduced serum/plasma vitamin C levels and an increase in intragastric pH in individuals with H. pylori infection (Woodward et al., 2001). Elevated intragastric pH levels can irreversibly denature ingested vitamin C in the stomach before it reaches the small intestine for absorption. A study by Mowat et al. investigated the effect of a 40 mg dose of omeprazole administered over one month on the concentration of vitamin C in the gastric juice of healthy individuals. The results showed that PPI therapy significantly decreased the concentration of vitamin C from 5 μm/L to 3 μm/L (Mowat et al., 1999). Similarly, the use of nonsteroidal anti‐inflammatory drugs (NSAIDs) can inhibit the uptake of vitamin C into leukocytes, affecting vitamin C stores in the body. In a randomized, double‐blind, parallel‐arm trial involving healthy men and women, the consumption of 2400 mg of aspirin for six days resulted in decreased vitamin C levels in urine, plasma, and particularly the gastrointestinal (GI) mucosa (Schulz et al., 2004). Additionally, aspirin increases the fecal excretion of vitamin C, hindering its absorption in the GI tract. Similar findings were observed in human and guinea pig models, where simultaneous oral administration of vitamin C with aspirin led to a decrease in drug concentration in plasma, leukocytes, and urine, accompanied by increased fecal excretion of vitamin C (Basu, 1982).

### 4.2 Effect of oral contraceptives (OCs) on the pharmacokinetics of vitamins

OCs decrease the serum levels of most vitamins, such as folic acid, B12, B6, B2, vitamin C, and vitamin E (Palmery et al., 2013). The use of OCs negatively impacted the folate status in the body, and mean serum levels were significantly decreased during the period of treatment, although folate levels were maintained after cessation of treatment (Shojania et al., 1968). In addition, OCs trigger the malabsorption of folate polyglutamate and increase the metabolism and excretion of folate in urine (Shojania, 1982). OCs containing estrogen lowered vitamin C levels in platelets and leukocytes as they increased the metabolism rate of this vitamin (Veninga, 1984). The use of inhaled corticosteroids is associated with decreased vitamin D and calcium levels, thereby increasing the possibility of bone fractures (Halpern et al., 2004; Weatherall et al., 2008).

### 4.3 Effect of pregnancy on the regulation of vitamin levels

Pregnancy demands an increase in energy and nutrition supply to keep the mother and fetus healthy. Nutritional deficiency during pregnancy is quite common in situations such as inadequate intake, malabsorption, dietary taboos, and increased fetal accumulation (Ladipo, 2000). Serum vitamins C and D levels dropped by 50% during pregnancy, partially due to increased fetal absorption by the adult fetus relying on maternal vitamin intake and partly due to hemodilution. Maternal serum vitamin D(25(OH)D) is low because of maternal plasma volume expansion, increased placental demand, increased vitamin D metabolism, and fetal development (Takaoka et al., 2020). These maternal vitamin levels significantly decreased from the first to the third trimester due to increased vitamin accumulation across the placenta (Lykkesfeldt & Tveden‐Nyborg, 2019; Tveden‐Nyborg & Lykkesfeldt, 2013). Similarly, vitamin A levels are lower with increasing gestational age, mostly reaching the lowest level during the third trimester. High fetal consumption and increased plasma volume are some possible reasons (Bastos Maia et al., 2019). Thiamine, riboflavin, niacin, and vitamin B6 levels in the blood also decrease during pregnancy, which is due to physiologic changes caused by increased blood volume or increased active transport through the placenta while increasing urinary excretion of their metabolites (Ladipo, 2000). Folate, vitamin B12, biotin, and pantothenic acid concentrations diminish due to poor intestinal absorption, insufficient intake, or increased demand during pregnancy (Milman, 2012).

### 4.4 Vitamin–vitamin interaction affecting the absorption of vitamins

Fat‐soluble vitamins can potentially interact in the intestines, although limited studies have been reported. An in vitro study of the uptake efficiency of one vitamin in the presence of other vitamins using Caco‐2 cells demonstrated that vitamin A absorption was not affected by either vitamin D or K (Goncalves et al., 2015). However, at medium and high doses, vitamin E considerably enhanced vitamin A absorption (up to 40%). When combined with vitamin A, vitamin E functions as an antioxidant in the proximal gut, preserving vitamin A. As a result, some of the vitamin E in the bolus was destroyed, resulting in decreased absorption in the distal region of the colon and reduced tocopherol plasma levels (Moore, 1940).

Vitamins A and D decreased vitamin E absorption in a dose‐dependent manner, while vitamin K had a detrimental effect only at the highest dosage. All fat‐soluble vitamins significantly reduced the absorption of vitamin K, ranging from 34% to 58%. Furthermore, vitamins E and K share similar metabolic pathways, and their interactions can lead to abnormal coagulation. Several possible mechanisms have been proposed for this interaction, including competition for an unknown enzyme that truncates the K1 side chain to form menadione, an increase in the metabolism and excretion of vitamin K as vitamin E activates xenobiotic pathways, and competition between vitamin E and K1 for CYP450, which hydroxylates the tail, thereby preventing the oxidation of the tail to form menadione (Traber, 2008).

### 4.5 Effect of pharmaceutical dosage forms on the bioavailability of vitamins

The absorption profiles of five different vitamin B12 formulations, i.e., nanoparticles (oral buccal spray), emulsions, standard tablets, chewable tablets, and liposomes (oral spray), were investigated. Throughout the clinical trials, the nanoparticle formulation demonstrated the greatest fast and sustained rise in vitamin B12 blood serum concentrations. Furthermore, when compared to all other equivalent dosage formulations supplied regardless of the method of administration, blood levels of vitamin B12 increased substantially from baseline to 1 and 3 h for just the nanoparticle‐delivered platform. The study concludes that nanoparticles can bypass the GI tract when delivered via the oro‐buccal tract, which gives direct access to facial lymphatics and allows them to pass into the systemic circulation; enhancing bioavailability (Vitetta et al., 2018). Fat‐soluble vitamins are inadequate in patients with cystic fibrosis (CF). Despite using several multivitamin tablets, CF patients still suffer from the inadequacy of these vitamins, presenting a need for new delivery technologies to enhance bioavailability. In a recent study, Nowak et al. formulated fat‐soluble vitamins in the form of liposomes and cyclodextrins and compared them to medium‐chain triglycerides (MCT) targeted for CF patients. Vitamin A formed into liposomes augmented the bioavailability of serum all‐trans‐retinol, and cyclodextrins enhanced the serum concentration of vitamin D3 and vitamin E relative to MCT (Nowak et al., 2021). When given orally, vitamins present low bioavailability, irregular absorption profiles, and high patient variation. Degradation, chemical instability, low GI absorption, and low water solubility contribute to the low oral bioavailability of vitamins. Hsu et al. (2019) discussed the use of lipid nanocarriers to improve bioavailability via oral delivery of vitamins. The ability of lipids in these nanocarriers to undergo lymphatic transport, bypass first‐pass metabolism, present high solubility due to lipid inclusion, increase the GI tract residence time, and inhibit P‐gp efflux from the intestine, ultimately increasing the oral bioavailability profile (Hsu et al., 2019; Managuli et al., 2018; Negi et al., 2013).

### 4.6 Influence of pharmacokinetics of vitamins through cellular mechanism

The most common route of vitamin administration is oral, even though parenteral administration is not infrequent. A detailed understanding of the mechanisms and factors affecting the absorption of vitamins is important to treat or prevent vitamin deficiency or insufficiency. Although not fully understood, the absorption of vitamins is affected by factors such as the mode of administration, dosage forms, administration dose, and site of absorption. In addition, several other conditions, such as defects in the intestinal uptake system, expression of transporters, colon‐related diseases, intestinal resection, chemical instability, and drug–drug interactions, might influence the absorption of vitamins, thereby reducing the bioavailability of vitamins (Hsu et al., 2019). It has been reported that the dietary level of vitamins regulates the intestinal absorption of ascorbic acid. According to Amano et al., supplementation of diet without vitamin C to animal models that cannot synthesize ascorbic acid showed enhanced expression of several transporters in the small intestine, suggesting a prominent role of intracellular ascorbic acid in the upregulation of sodium‐dependent vitamin C transporters (Amano et al., 2010). Intestinal uptake of dietary folates depends on the food matrix entrapping folates, intestinal folylpolyγ‐glutamate carboxypeptidase inhibitor, stability of reduced folates in the intestinal microenvironment, and so on (Elnakat & Ratnam, 2004). Therefore, homeostasis of vitamins is of clinical and nutritional significance, especially vitamins that cannot be sufficiently synthesized within the human body depending on intake from exogenous sources and intestinal uptake (Said, 2011).

### 4.7 Pharmacokinetics of vitamin B1 through cellular mechanisms

**FIGURE 3:**
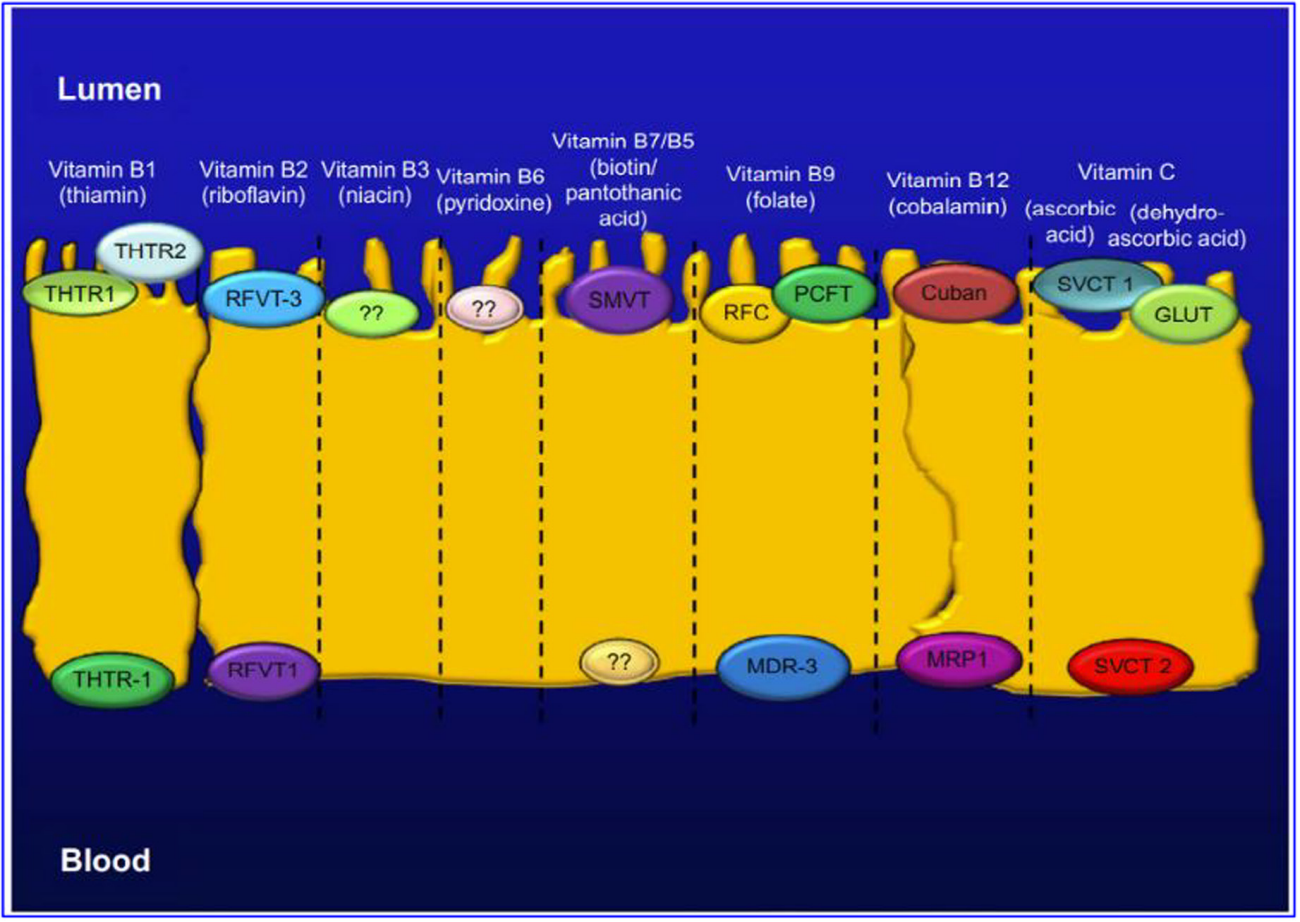
Diagrammatic representation showing membrane transporters involved in the absorption of dietary water‐soluble vitamins in the small intestine. Reprinted with permission from Said and Nexo (2018). Copyright. SMVT stands for sodium‐dependent multivitamin transporter; RFC stands for reduced folate carrier; MDR‐3 stands for multidrug‐resistance protein‐3; PDZD11 stands for PDZ‐containing protein 11; RFVT‐1, RFVT‐3, THTR‐1, THTR‐2, and TPP stands for thiamine pyrophosphate. Niacin, pyridoxine, and TPP transporters have all been identified based on their functions, but their molecular identities are unknown. The accessory protein PDZD11 interacts with SMVT and has an impact on both the cell biology and function of SMVT. Dashed lines separate transporters for different vitamins from one another.

Vitamin B1 is not endogenously synthesized by humans and is stored in the body for a relatively short time before excretion, so the required nutritional value is obtained from dietary and bacterial sources (Smithline et al., 2012). Dietary thiamine in its phosphorylated form as thiamine pyrophosphate, a coenzyme form of vitamin B1 is hydrolyzed in the intestinal lumen into free thiamine, which enters the intestinal membrane via active transport, a specific pH‐dependent and sensitive carrier‐mediated mechanism (Said, 2011). Remarkably, thiamine transport is enhanced by the pH gradient at low concentrations but involves passive diffusion at higher doses (Rindi & Laforenza, 2000). In mammals, thiamine is taken up by two human intestinal transporters of thiamine, namely, hTHTR‐1 and hTHTR‐2 (Figure 3) (Kunisawa, 2016; Said & Nexo, 2018). Intestinal uptake of thiamine is regulated by intracellular calcium/calmodulin‐dependent pathways, and its insufficiency leads to the induction of transporter‐mediated pathways, especially hTHTR‐2 (Said, 2011). However, chronic consumption of alcohol caused thiamine deficiency inhibition of intestinal thiamine uptake with a marked decrease in the level of hTHTR‐1 but not hTHTR‐2 (Langlais, 1995). Smithline et al. (2012) studied the pharmacokinetic profile of oral thiamine hydrochloride in human subjects and demonstrated that thiamine can be absorbed by active and unsaturated passive transport up to 1500 mg. In addition, lower administration doses of thiamine showed a significant role in active transport compared to higher doses. However, this study did not include the tissue distribution of thiamine.

### 4.8 Pharmacokinetics of vitamin B2 and B6 through cellular mechanisms

Another two vitamins that are obtained from dietary and intestinal microflora are pyridoxine and riboflavin (both vitamins are absorbed from the intestinal membrane). Recent mechanism‐based studies on the intestinal absorption of pyridoxine demonstrated the role of an acidic pH‐dependent carrier‐mediated system rather than the involvement of a sodium‐dependent carrier mechanism (Said et al., 2003). However, intestinal uptake of pyridoxine is highly regulated by several extracellular and intracellular factors, such as low vitamin levels (upregulation), increased cAMP levels (inhibition), and the PKA‐mediated pathway (Said et al., 2003, 2008). Riboflavin in the diet exists in free form and flavin mononucleotide (FMN) and flavin adenine dinucleotide (FAD) forms, where FMN and FAD forms are converted to free form by intestinal phosphatases (Daniel et al., 1983). Riboflavin absorption‐based mechanistic studies demonstrated the involvement of a sodium‐independent carrier system. Two riboflavin‐based transporters, RFT1 and RFT2, are widely expressed in the human intestinal membrane, where transporter 2 appears to be more efficient than transporter 1. Simultaneously, riboflavin‐based transporter 3 (RFT3) was identified and reported as a brain‐specific transporter, and it has recently been shown to be involved in the apical uptake of riboflavin by intestinal epithelial cells (Yao et al., 2010; Yoshimatsu et al., 2014).

### 4.9 Influence on the pharmacokinetics of vitamin B7 through cellular mechanisms

Similar to other water‐soluble vitamins, biotin is exposed to the intestinal membrane from two sources: exogenous sources (diet) and normal intestinal microflora, even though the role of bacterial sources in total body biotin is unclear (Dasgupta, 2019). Biotin exists as free biotin or protein‐bound biotin in the diet. The latter form is digested by proteases and peptidases to biocytin and short peptide biotin conjugates, which are further converted to free biotin by the enzyme biotinidase before absorption (Wolf et al., 1984). The intestinal absorption mechanism of biotin studied in humans and other mammals reported the involvement of sodium‐dependent carrier‐mediated transporters present in the small and large intestines. This transporter system is also involved in the translocation of pantothenic acid and lipoate and is hence referred to as the sodium‐dependent multivitamin transporter (SMVT) (Said, 2011; Vadlapudi et al., 2012). Previously, a group of researchers studied the transporter function of cationic histidine residues in hSMVT to transport anionic biotin, which exhibited a significant role of histidine 115 and histidine 254 (out of seven histidine residues across species) in carrier‐mediated biotin uptake (Ghosal & Said, 2011).

### 4.10 Pharmacokinetics of vitamin B9 through cellular mechanisms

**FIGURE 4:**
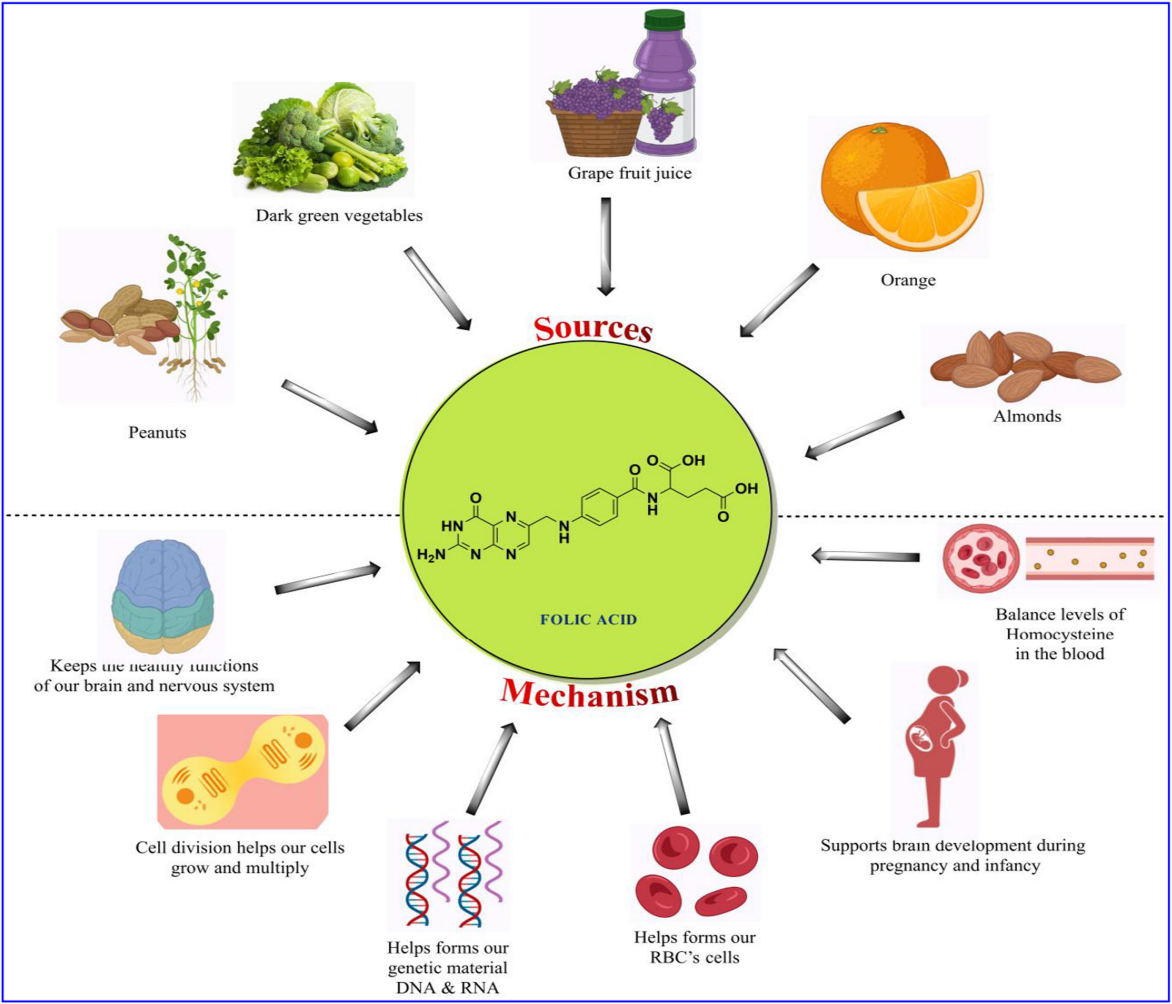
Source of folic acid and its effect on cellular mechanisms.

Another vital vitamin is folate, which cannot be synthesized in humans and mammals but is achieved by the host either from a dietary source or synthesized in the colon by normal microflora. Polyglutamate forms of dietary folates are hydrolyzed to the mono‐glutamate forms and are transported across the intestinal membrane via a pH‐dependent carrier‐mediated pathway (Visentin et al., 2014). Folates are transported mainly by two facilitative solute carriers, a reduced folate carrier (RFC or SLC19A1) and a proton‐coupled folate transporter (PCFT or SLC46A1) (Figure 4) (Said & Nexo, 2018). RFC is the major transporter that is expressed in almost all human and murine cell lines and helps transport folates to systemic tissues under physiological conditions. In addition, this transporter is expressed in the intestinal apical brush‐border membrane, proximal renal tubule basolateral membrane, choroid plexus apical membranes, and retinal pigment epithelium (Zhao et al., 2011; Zhao & Goldman, 2013). Next, the transport of folates through the PCFT transporter is higher at acidic pH and has an affinity for both folic acid and reduced folate (Qiu et al., 2006). PCFT is also expressed in most tissues as RFC along with the sinusoidal membrane of the liver and placenta and has been shown to play a crucial role in the intestinal transport of folate (Gnana‐Prakasam et al., 2011; Qiu et al., 2006; Urquhart et al., 2010; Zhao et al., 2009). Folate receptors that are expressed on the cell surface and anchored in the cell membranes by a glycosylphosphoinositol domain have a high affinity for folic acids. In particular, FR_α_ and FR_β_ are responsible for the internalization of receptor‐bound folates and their conjugates (Elnakat & Ratnam, 2004). Intestinal uptake of folate is also regulated intracellularly by protein tyrosine kinase (PTK) and cAMP‐mediated pathways, whereas folate deficiency upregulates epithelial uptake (Kumar et al., 1997). Moreover, human pancreatic cellular uptake of folates involves a pH‐dependent carrier‐mediated mechanism and is also regulated by intracellular PTK and cAMP (Nabokina et al., 2004).

Folate is the natural form of vitamin B9, which is found naturally in many foods, such as peanuts, dark green vegetables, grapefruit juice, orange fruits, and almonds. It is crucial during periods of fast cell and tissue growth, such as infancy, puberty, and pregnancy, since it helps produce DNA and RNA, the body's genetic material. Additionally, it helps to support brain and nervous system development. Oral supplementation with folic acid encourages the development of platelets, white blood cells, blood vessels, and red blood cells in those with certain megaloblastic anemias.

### 4.11 Influence of the pharmacokinetics of vitamin B12 through cellular mechanisms

The absorption of cobalamin (Cbl) in the body is mediated by a set of sophisticated processes. Cbl in the diet is captured by haptocorrin present in the upper GI tract, which is degraded by pancreatic enzymes in the duodenum to release the vitamin. Cbl forms a complex with intrinsic factor (IF) in gastric fluid, followed by internalization of the complex by absorptive cell receptors (cubilin) present in the distal ileal mucosa via receptor‐mediated pathways (Fedosov, 2012; Quadros, 2010). IF is degraded by lysosomes and liberates Cbl, which transverse the lysosomal membrane to enter the cytoplasm. Next, it enters the systemic circulation and is transferred to plasma transcobalamin. This plasma protein delivers vitamins throughout the body tissues (see detailed review) (Nielsen et al., 2012). During early development, transcobalamin (TC) receptors on the apical membrane of the human intestine might mediate the uptake of Cbl by transcytosis (Bose et al., 1997).

### 4.12 Pharmacokinetics of vitamin C through cellular mechanisms

**FIGURE 5:**
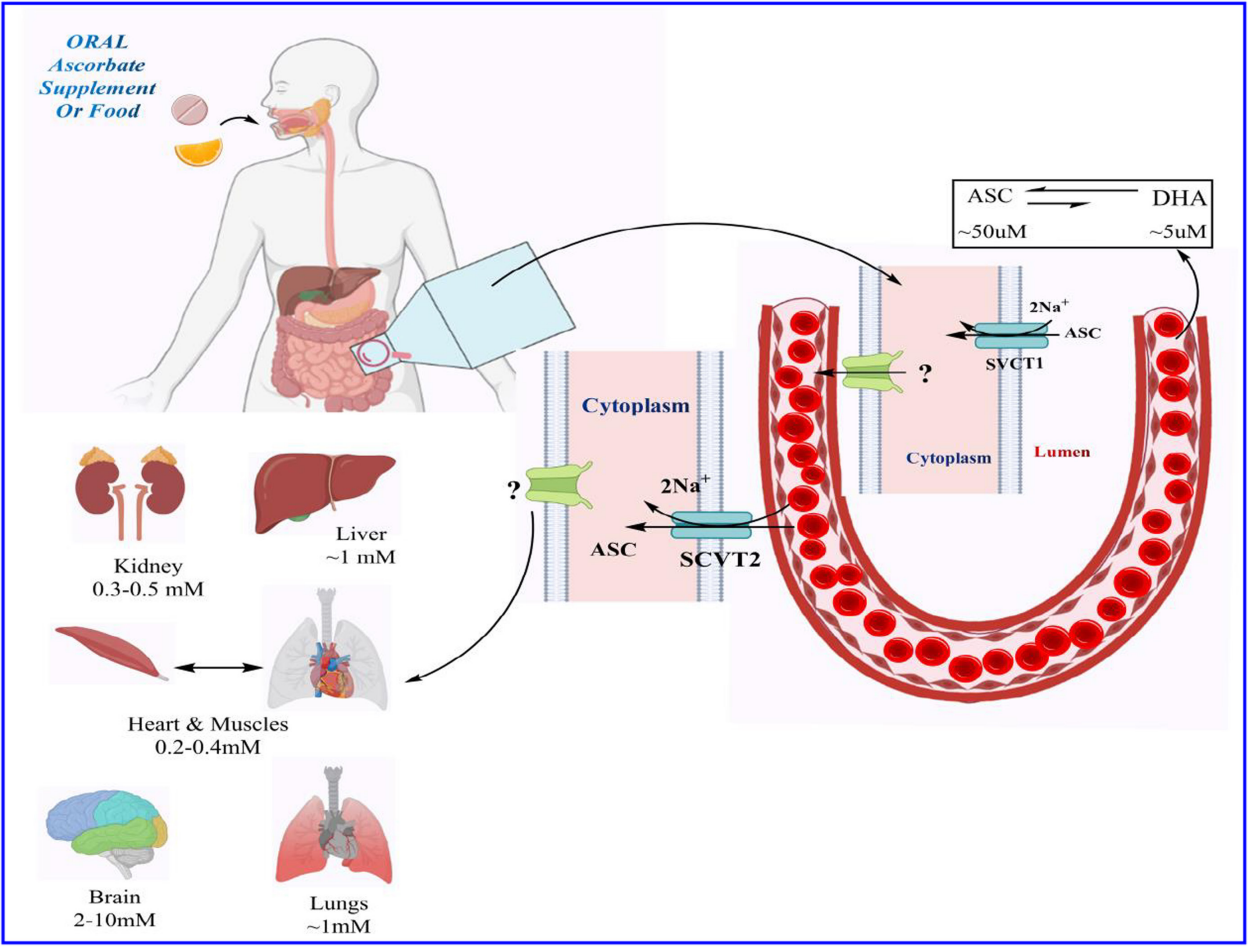
The absorption of vitamin C in the intestines, its distribution in tissues, and its reuptake in the kidneys are all regulated by sodium‐dependent vitamin C transporters.

Vitamin C exists as ascorbate (reduced form) or dehydroascorbic acid (DHAA, oxidized form), the former being predominant in most biological fluids (Rose, 1987). The pharmacokinetics of vitamin C are complex, and most of the intestinal absorption, tissue distribution, and reuptake in the kidney is governed by sodium‐dependent vitamin C transporters (SVCT) (Figure 5) (Lindblad et al., 2013; Wilson, 2005). Although absorption of vitamin C occurs via passive diffusion, facilitated diffusion, and active transport, the predominant anionic form at neutral pH prevents passive diffusion across the biological membrane. In addition, the ionized form in the stomach (99.9%) and small intestine (15%) plays a significant role in passive uptake (Lykkesfeldt & Tveden‐Nyborg, 2019). Intestinal transport studies performed in the human brush border membranes displayed absorption of both ascorbates and DHAA along the entire human intestinal membrane, with discrepancies in tissue specificity, absorption kinetics, and energy dependence (Rumsey & Levine, 1998). In particular, intestinal uptake of ascorbate is sodium‐dependent, potentially sensitive, and saturable (saturated with increasing concentrations of glucose), whereas DHAA facilitates diffusion (Malo & Wilson, 2000). Two SVCTs, namely, SVCT‐1 (598 amino acids) and SVCT‐2 (650 amino acids), are expressed in the apical and basolateral membranes of the intestine and show relatively lower selectivity for D‐ascorbic acid than for L‐ascorbic acid (Boyer et al., 2005; Tsukaguchi et al., 1999). In addition, glucose transporters (GLUT1, GLUT3, and GLUT4) are involved in the intestinal absorption of DHAA (Said & Mohammed, 2006). Additionally, active transport of ascorbate across the luminal membrane of the renal proximal tubule plays a crucial role in bioavailability (Malo & Wilson, 2000). Interestingly, approximately 90% of the total vitamin C is absorbed when the daily human intake is below 100 mg; however, the absorption efficiency decreases with increased intake (Naidu, 2003). A study by Padayatty et al. showed that the plasma concentration of vitamin C is tightly controlled when administered through the oral route compared to intravenous administration (Padayatty et al., 2004).

Oral administration of vitamin C has been absorbed primarily by either membrane transporters in the apical brush border membrane, as dehydroascorbic acid (DHA) through facilitated diffusion via GLUT1 or GLUT3 transporters or as ascorbate (ASC) by sodium‐coupled active transport via the SVCT1 transporter. Once within the cell, GLUT1 and GLUT2 in the basolateral membrane effectively convert DHA to ASC or transport it to circulation, maintaining a low intracellular concentration and promoting additional DHA absorption. ASC is transported to plasma through diffusion; the particular efflux mechanisms are yet unclear. Diffusion may potentially be promoted by volume‐sensitive anion channels or by active transporters that have not yet been discovered. When the supply of vitamin C is insufficient, certain organs maintain high levels at the expense of other organs through concentration‐dependent processes. The brain is particularly protected. Additionally, the concentration‐dependent processes of absorption and reabsorption play a role in the body's homeostatic regulation of vitamin C. Vitamin C is effectively filtered through the renal tubule lumen in the kidney by the glomerulus. Under vitamin C deficiency circumstances, SVCT1 transporters in the apical membrane are highly responsible for reabsorption; however, diffusion from the luminal surface may also play a role in total uptake. ASC is released into the bloodstream by diffusion, similar to in the intestinal epithelium, although the full amount and mechanisms of this remain unknown. DHA can be transported to plasma through GLUT2 transporters that are found in the basolateral membrane. Vitamin C is excreted quantitatively under saturated circumstances.

### 4.13 Fat‐soluble vitamins

**FIGURE 6:**
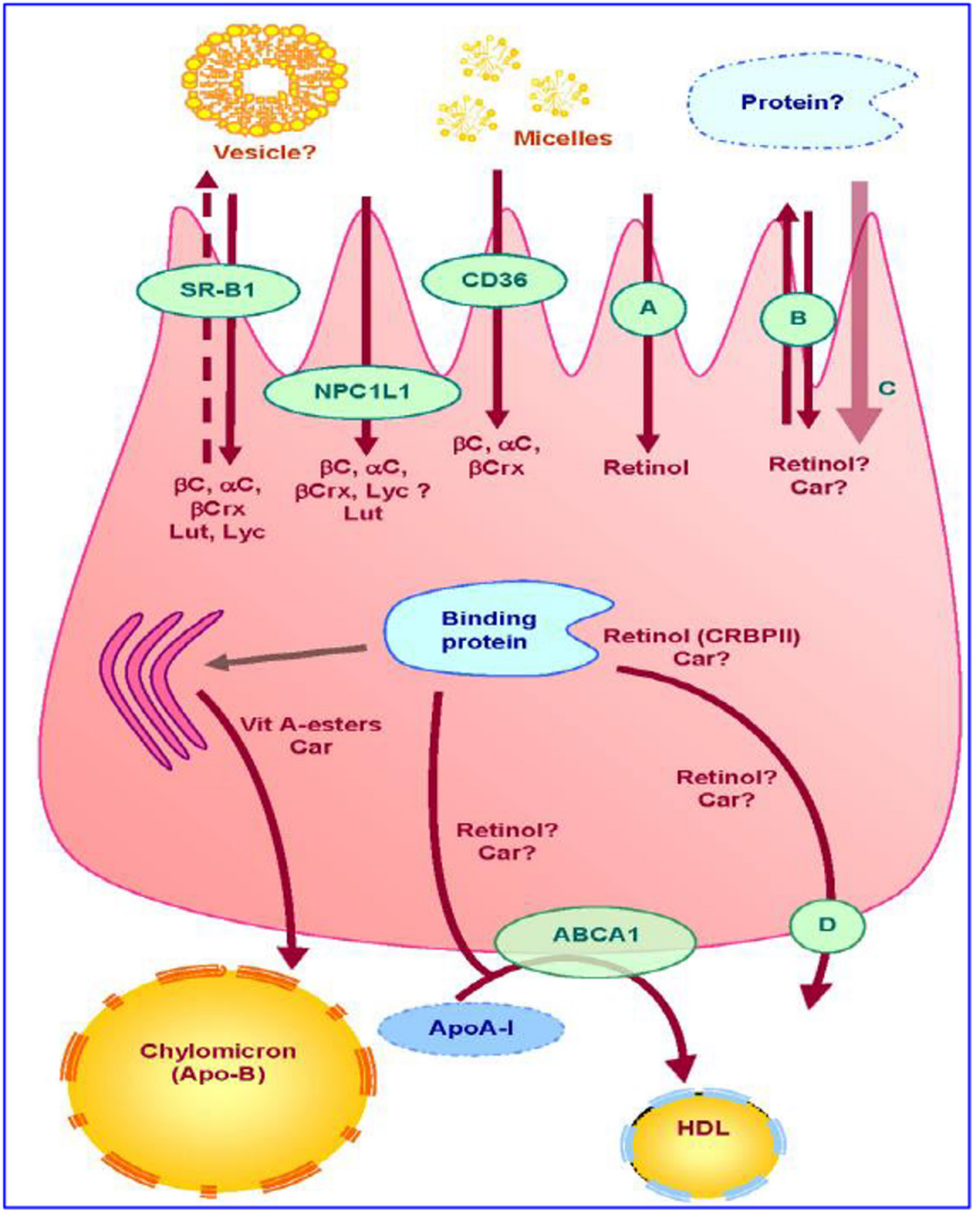
Proteins involved in uptake, transport, and secretion pathways of vitamin A and carotenoids across the enterocyte. ?, putative pathway; A, retinol putative specific transporter; B, unidentified apical transporter; C, passive diffusion; Car, carotenoids; D, unidentified basolateral efflux transporter; Lut, lutein; Lyc, lycopene; Vit, vitamin; αC, α‐carotene; βC, β‐carotene; βC, β‐cryptoxanthin. Carotenoids are captured from mixed micelles by apical membrane transporters: SR‐BI, CD36, and NPC1L1.

The absorption of fat‐soluble vitamins closely follows pathways similar to those of other dietary fats and requires bile acids to emulsify the lipids. Dietary vitamin A is obtained from animal sources as retinyl esters and plant sources as provitamin A carotenoids (Penniston & Tanumihardjo, 2006). Prior to absorption from the intestinal lumen, most retinyl esters are hydrolyzed by pancreatic and brush border retinal ester hydrolase into retinol and free fatty acids; however, carotenoids are converted to retinol by retinal reductase (Rigtrup & Ong, 1992). Interestingly, retinol obtained from both sources is further esterified into retinyl esters, which are incorporated with chylomicrons before secretion into the lymphatic system (Nayak et al., 2001; Penniston & Tanumihardjo, 2006; Reboul, 2013). Prior studies also suggested the absorption of unesterified retinol via multiple mechanisms (facilitated and passive diffusion) at physiologic concentrations (Harrison, 1821). An intestinal uptake study performed in mutant mouse models demonstrated a significant role of scavenger receptor class B (SR‐B1) in facilitating the absorption of β‐carotene (van Bennekum et al., 2005). In addition, Reboul et al. (2009) demonstrated that the presence of basolateral cholesterol transporter ATP‐binding cassette, subfamily A, member 1 (ABCA1) enables the absorption of retinol in enterocytes. At higher vitamin A concentrations, it is transported via simple passive diffusion. Cellular retinol‐binding protein type II (CRBPII) is also expressed in the intestinal mucosa and facilitates retinol absorption from dietary sources (D'Ambrosio et al., 2011; Uchio et al., 2002). Before intestinal absorption, the dissolution of vitamin D in the lipophilic content of the food takes place, forming a mixed micelle, and their absorption efficiency significantly depends on the percentage of vitamin encapsulation (Maurya & Aggarwal, 2017). Absorption of non‐hydroxylated forms of vitamin D, specifically vitamin D_2_ and D_3_, is facilitated by a passive diffusion mechanism at higher concentrations, whereas carrier‐dependent proteins are mediated at low dietary concentrations (Reboul et al., 2011). Similar to vitamin A, intestinal absorption of vitamin D and vitamin E is facilitated by SR‐B1 along with other cholesterol transporters, such as cluster determinant 36 (CD 36) and Neimann‐Pick C1‐Like 1 (NPC1L1) (Maurya & Aggarwal, 2017). However, the incorporation of vitamin D into the micelles and intestinal uptake is impaired by the copresence of α‐tocopherol and phytosterol (Goncalves et al., 2011; Reboul et al., 2011). Previous studies have reported that vitamin E is absorbed from the upper half of the small intestine. However, recent findings highlighted the distal jejunum and ileum as the main parts responsible for absorption (Goncalves et al., 2015; Reboul & Borel, 2011). In addition, Porsgaard and Hard demonstrated dissimilar intestinal absorption between stereoisomers of vitamin E, with α‐tocopherol showing greater absorption than γ‐ and δ‐tocopherols, which might be due to the metabolism of the latter two stereoisomers by ω‐hydroxylase (Bardowell et al., 2012; Traber et al., 1990). Like several other vitamins, vitamin K is obtained from exogenous sources (dietary and microflora), and intestinal absorption plays a major role in regulating vitamin K homeostasis (Booth, 2012; McKeown et al., 2002). Absorption of vitamin K1 from the proximal part of the small intestine is carrier‐mediated, energy‐dependent, and highly affected by the presence of bile acids. However, studies have suggested that the presence or absence of medium‐chain and long‐chain fatty acids does not impact their absorption (Shearer et al., 1975). However, the passive diffusion of vitamin K2 is affected by the change in the concentration of bile acid and unsaturated fatty acids along with absorption site pH (Hollander et al., 1977). Recent in vivo studies in rodent models revealed cholesterol transporter (NPC1L1) as a key regulator in mediating intestinal absorption of vitamin K1 (Tappei et al., 2015). Overall, fat‐soluble vitamins demonstrate competition for intestinal absorption, which might be due to common absorption pathways (Goncalves et al., 2011). Apical membrane proteins involved in apical uptake of retinol have not yet been identified. A fraction of vitamin A and carotenoids may then be effluxed back to the intestinal lumen via apical membrane transporters (SR‐BI and possibly other transporters) (Figure 6). Another fraction is transported to the site where they are incorporated into chylomicrons. Some proteins may be involved in the intracellular transport of carotenoids, but none have been identified. Conversely, CRBPII has clearly been described as being involved in the intracellular transport of retinol. Retinyl esters and carotenoids are secreted in the lymph into chylomicrons, while some of the more polar metabolites may be secreted by the portal route. It is suggested that free retinol can also be secreted at the basolateral side via ABCA1 (apoAI pathway).Open access from MDPI (Carr et al., 2017).

## 5 NANOMEDICINE INFLUENCES THE PHARMACOKINETICS OF VITAMINS IN A POSITIVE MANNER

In pharmaceuticals, formulation technology has grown dynamically and achieved extensive acceptance due to improved chemical stability, targeted tissue distribution, controlled drug release, and enhanced bioavailability of drugs (Fidaleo et al., 2021). The development of vitamin‐loaded microemulsions for oral delivery of vitamins A and E demonstrated a significant increase in intestinal absorption and oral bioavailability (Julianto et al., 2000; Taha et al., 2009). This might be attributed to improved lipophilicity upon encapsulation into the formulation as well as surfactant‐induced alteration in intestinal membrane and fluidity (Hsu et al., 2019). To enhance the oral bioavailability of vitamin B12, Sarti et al. developed a poly(acrylic acid)‐cysteine (PAA‐cys)‐based formulation, which demonstrated an approximately 4‐fold increase in *the* ex vivo absorption across rat intestinal mucosa and a 3‐fold increase in oral bioavailability (Sarti et al., 2013). A recent study compared the absorption profiles of 5 diverse formulations of vitamin B12 in healthy subjects administered through different oral delivery platforms (Vitetta et al., 2018). Nanoparticles (NanoCelle) containing methylcobalamin B12 administered as an oral‐buccal spray at the dose of 2000 μg/600 μL/2 dose demonstrated significantly higher absorption compared to all other equivalent doses from tablets (oral and sublingual), emulsions (sublingual), or liposomes (oral spray). However, the nanoparticles of B12 showed bioequivalence with a dissolvable tablet containing a fivefold higher dose of vitamin B12, which suggested an important role of formulation and administration route on the absorption of vitamins. Similarly, an oral liposomal formulation of vitamin C was developed using >20% w/w lipids. The mean diameter of the prepared liposomal vitamin C was determined to be below 200 nm, as measured using dynamic light scattering and cryo‐TEM. In addition, pharmacokinetic profiles after oral administration of vitamin C‐loaded liposomes in healthy volunteers (n = 20) demonstrated an increase in the concentration maximum (C_max_) and time to reach maximum plasma concentration (T_max_) as well as enhanced oral bioavailability compared to aqueous solution (Łukawski et al., 2020). In addition, the incorporation of vitamin C inside lipid‐based formulations maintains high vitamin levels in the intestinal tract and prevents vitamin C degradation without compromising potency at the cellular level. An open‐labeled, randomized, single‐dose study using a liposomal formulation of vitamin C with a particle size below 100 nm and an encapsulation efficiency of approximately 65% also demonstrated 1.77‐fold higher oral bioavailability compared to that of nonliposomal vitamin C, demonstrating a lipid‐based drug delivery strategy as a potential platform to deliver hydrophilic drugs (Gopi & Balakrishnan, 2021).

### 5.1 Enhancement of the pharmacokinetic effect of hydrophobic drug moieties by using nanoformulation techniques

To overcome the absorption challenges of hydrophobic drugs, several formulation strategies can be applied, such as solid crystalline formulations (salt formation and micronization), amorphous formulations (spray drying and melt extrusion), and lipid‐based formulations (solid dispersions, solid lipid nanoparticles, emulsions, liposomes, etc.) (Pouton, 2006). In addition, lipid‐based formulations minimize the inherent limitations of hydrophobic drugs by improving dissolution profiles and enhancing absorption. In addition, these formulations demonstrate reproducible pharmacokinetic profiles in terms of C_max_ and T_max_ (Łukawski et al., 2020). Therefore, to enhance the oral bioavailability of fat‐soluble vitamin A, Taha et al. prepared a self‐nanoemulsified drug delivery (SNEDD) formulation filled in capsules (F1) and a SNEDD formulation compressed as tablets (F2) and further performed a comparative bioavailability assessment in rats. The investigation showed that vitamin A‐loaded F1 and F2 enhanced the rate and extent of absorption compared to the control solution (oily drug solution) (Taha et al., 2007). Moreover, the oral bioavailability of F1 and F2 was 2‐fold and 1.4‐fold greater than that of the control, respectively, suggesting a promising role of the emulsified delivery system in enhancing absorption. Vitamin D3 orodispersible film (ODSF) that dissolves in the mouth of healthy subjects exhibited comparable bioavailability with that of an equivalent dose of marketed oral vitamin D3 solution (Radicioni et al., 2022). Although vitamin E is marketed as soft capsules and tablets, it possesses low oral bioavailability. Therefore, different nanoformulation strategies, including solid‐lipid nanoparticles, nanovesicles, lipid carriers, and polymeric nanoparticles, have been applied to address oral bioavailability issues. In a study, the complexation of vitamin E with γ‐cyclodextrin improved the solubility as well as the concentration of plasma and tissue tocotrienol by increasing intestinal transport, thereby enhancing oral bioavailability (Ikeda et al., 2010). Similarly, solid lipid nanoparticles of γ‐tocotrienol increased the in situ intestinal absorption by 10‐fold compared to the γ‐tocotrienol micellar solution (Abuasal et al., 2012). Vitamins such as biotins, vitamin B12, and folates have also been used as ligands for drug delivery to target receptors in the intestinal epithelia (Rindi & Laforenza, 2000; Said, 2011). Functionalization of liposomes with thiamine or niacin and nanoparticles with vitamin B12 facilitated the interaction with vitamin receptors, thereby enhancing the oral absorption of both insulin‐loaded formulations (He et al., 2018; Verma et al., 2016). Moreover, the incorporation of folic acid as a targeting ligand grafted in nanoparticles assisted the absorption of vancomycin and insulin via pH‐dependent and sodium‐dependent transport mechanisms (Chen et al., 2022).

### 5.2 Absorption and distribution of vitamins

Water‐soluble vitamins can be absorbed into systemic circulation for immediate tissue use, whereas absorption of lipid‐soluble vitamins mainly depends on the intestinal lymphatic transport that facilitates access to the systemic circulation. Most orally administered drugs gain access to the systemic circulation through passive/active uptake by enterocytes, enter the portal vein via mesenteric vessels, and then undergo hepatic first‐pass metabolism. However, some highly lipophilic drugs, such as log D_7.4_ >5 and triglyceride solubility >50 mg/mL, have the potential to be absorbed into intestinal lymphatics following oral coadministration with lipids (Charman & Stella, 1986). Briefly, dietary lipids are hydrolyzed into free fatty acids and 2‐mono glycerol to form micelles by bile salts and pancreatic lipase in the lumen of the intestine, allowing lipophilic vitamins to diffuse through the unstirred water layer and then across the enterocyte membrane. In enterocytes, triglycerides are resynthesized into chylomicrons (CMs) and simultaneously associated with lipid‐soluble vitamins. Although the mechanisms of the compound‐CM association process are not fully understood, it is clear that CMs are vital carriers that transport lipophilic compounds into the intestinal lymphatic system. The advantage of lipophilic compound‐CMs is that they bypass hepatic metabolism via intestinal lymphatic transport, and they improve pharmacokinetic performance (Qin et al., 2021; Zgair et al., 2016). Lipophilic prodrugs of lopinavir with high affinity for CMs lead to intestinal lymphatic transport with high concentrations of lopinavir in mesenteric lymph and mesenteric lymph nodes. The cumulative doses transported into the mesenteric lymph as well as oral bioavailability following oral administration of lipid‐soluble vitamins have been widely recognized. As an example, intestinal lymphatic absorption ranged from 35.6% to 41.6% of the total dose following infusion of vitamin A micellar solution in rats (Hollander & Dadufalza, 1990). Arik Dahan and Amnon Hoffman demonstrated that the cumulative amount of vitamin D3 that appeared in mesenteric lymph was approximately 33% of the dose following a single oral administration of 0.5 mg/kg in rats. For vitamin E, lymphatic absorption is thought to occur predominantly via enterocyte CM formation and secretion to deliver it to the lymphatic system (Rigotti, 2007).

It has been confirmed that vitamin C is more likely to accumulate in tissue cells than in the plasma of healthy humans following administration. More importantly, the retention time of vitamin C in organs is long‐lasting to achieve high levels during states of deficiency at the expense of other organs, especially the brain (Hasselholt et al., 2015; May, 2012; Tveden‐Nyborg et al., 2012). Similar to vitamin C, the concentrations of vitamin B in the brain also remain relatively high. For example, 5‐methyltetrahydrofolate levels in the brain were as high as 4‐fold compared to those in plasma (Spector & Lorenzo, 1975); moreover, it has been reported that there were approximately 50 times more biotin and pantothenic acid in the brain than in plasma (Spector & Johanson, 2007). Those high concentrations of water‐soluble vitamins B and C in the brain are of service to maintain proper brain function in daily life.

### 5.3 Metabolism of vitamins

Other than water‐soluble vitamins, lipid‐soluble vitamins possess different biodistributions in adipose tissues following administration. As mentioned above, those lipophilic vitamins are not absorbed directly into the portal vein to the systemic circulation but are absorbed into the intestinal lacteals via CMs, transported through the mesenteric lymphatic system to the bloodstream. In the circulation, CM vitamins can be delivered to peripheral tissues, such as adipose tissue and muscle. CMs are hydrolyzed by lipoprotein lipase to release and store vitamins in these tissues. The liver for further metabolism takes up the resulting chylomicron remnants. Therefore, lipid‐soluble vitamins are more likely to accumulate in body tissues, thereby leading to toxicity. For example, lipophilic vitamin 25(OH) D_3_ has a high affinity for fat, which is preferably stored in adipose tissues and occasionally redistributed into the bloodstream, with a long half‐life of 2 weeks to 3 months (Heaney et al., 2011; Jones et al., 2014; Oliveri et al., 2015; Spector & Johanson, 2007). Thus, vitamin D hormones may not require daily replenishment to avoid accumulation in adipose tissue, and the risk for toxicity begins to increase. In addition, drug–drug interactions are another reason that can reduce the bioactivation rate from vitamin D_3_ to 1,25‐dihydroxyvitamin D3(1,25(OH)_2_D_3_) and increase accumulation toxicity. It is well known that hepatic 25‐hydroxylase (CYP25) is the major enzyme responsible for bioactivation; however, HIV‐protease inhibitors such as ritonavir can inhibit cytochrome P450 enzymes, including CYP25, contributing to bone demineralization (Cozzolino et al., 2003; Oliveri et al., 2015). Therefore, plasma levels of vitamins should be monitored as a consequence of certain treatments that can either inhibit or induce the activity of metabolic enzymes in patients. Vitamins A and K are also stored in adipose tissues in the body, leading to toxicity to the liver and kidney from excessive intake of vitamin A, but the outcome toxicity from vitamin K is extremely rare (Booth, 2012; El‐Sohemy et al., 2001; Hathcock et al., 1990; Traber & Kayden, 1987). It has been reported that approximately 90% of the total dosed vitamin E can be stored in adipose tissues and last for a long time to clearance. Interestingly, studies on obesity have shown that vitamin E can effectively reduce fibrosis, inflammation, oxidative stress, and toxicity in adipose tissues in obesity‐related insulin resistance mouse models (Alcalá et al., 2015). Overall, the biopharmacokinetic profiles of lipid‐soluble vitamins will be mainly dependent on adequate digestion and absorption of dietary lipids, which is correlated with our previous statement that dietary lipids facilitate the lymphatic absorption of lipid‐soluble vitamins. However, excessive accumulation of lipid‐soluble vitamins in the body might cause health issues.

### 5.4 Excretion of vitamins

Excessive intake of water‐soluble vitamins is unable to cause toxicity in which they are readily excreted from the body. For example, high doses of vitamin C administration can immediately saturate plasma concentrations, leading to excessive vitamin C being excreted in the urine (Levine et al., 2011). In contrast to vitamin C, unabsorbed vitamin B12 is highly bound to bile or degraded in the intestine and then excreted in the feces (Allen, 2010; Teo et al., 1980). In addition, urinary excretion of vitamin B12 is another major pathway for elimination from the body; however, it has been reported that urinary excretion highly depends on urine volume rather than the intake dose of vitamin B12 (Fukuwatari et al., 2009; Kulkarni et al., 1976). The excretion of other vitamin B also occurs in both feces and urine (Schultz et al., 1938). Unlike vitamin B12, urinary excretion cannot be affected by physical characteristics, urine volume, and urinary creatinine in healthy subjects (Fukuwatari et al., 2009). Most lipid‐soluble vitamins are excreted as water‐soluble glucuronide metabolites in feces and urine; therefore, liver disease is usually associated with a significant decrease in the levels and rate of excretion (Shearer et al., 1970; Stumpf et al., 1981; Varma & Beaton, 1972). For example, patients living with alcoholic cirrhosis showed considerably slower plasma vitamin D clearance as well as a 36% decrease in total urinary excretion compared to healthy controls (Avioli et al., 1967).

## 6 TOXICITY OF VITAMINS

Within the United States, the FDA diligently oversees the effectiveness and safety of all pharmaceuticals through regular evaluations. Notably, the FDA does not mandate the submission of efficacy documentation for vitamins and supplements (Charen & Harbord, 2020; I.J. of H.S. and R. (IJHSR), 2018). Consequently, an alarming number of nearly 50,000 incidents of vitamin toxicity are reported annually to poison control centers in the US (American Association of Poison Control Centers, n.d.). According to data from the National Health and Nutrition Examination Survey (NHANES), spanning from 2017 to March 2020, a significant proportion of 34.8% of children and adolescents, along with 58.5% of adults, acknowledged the use of at least one dietary supplement within the past 30 days. Notably, utilization was higher among females than males, except for children aged 12 to 24 months (Gummin et al., 2017; Stierman et al., 2021). Table 2 reveals the source and toxicity of vitamins.

**TABLE 2:** Demonstrates the source and toxicity of vitamins.

Name	Food source	Toxicity	Ref.
A	Leafy vegetables, carrots, pumpkin, broccoli, and tomatoes.	Cirrhotic like liver syndrome, birth defects, and fatigue	Olson et al. (2023)
D	Tuna fish, dairy products, and eggs	Brain, cardiac, kidney damage, and calcification	Asif and Farooq (2023)
E	Sunflower seed‐oil, almonds, and peanuts	Allergic dermatitis, and impaired blood clotting	Owen and Dewald (2023)
K	Cabbage, broccoli, and green leafy veggies	Hemorrhagic diseases, and anemia	Mladěnka et al. (2022)
C	Lemon, kiwi, orange, potatoes, and onion	Diarrhea, and kidney stone	Abdullah et al. (2023)
B1	Cereals, lentils, pork, beans	Vasodilation, and cardiac arrhythmias	Martel et al. (2022)
B2	Cheese, eggs, yogurt, and salmon	Abnormal liver function and jaundice	Peechakara and Gupta (2022)
B3	Red meat, fish, nuts, and bananas	Diarrhea, and insulin resistance	Mazur et al. (2022)
B5	Pees, potatoes, chicken breast, nuts, and milk	Tingling feet, nausea, and heartburn	Sanvictores and Chauhan (2023)
B6	Spinach, sunflower seeds, salmon tuna	Sensory neuropathy, seizures	Hemminger and Wills (2023)
B7	Onion, grains, eggs, salmon, and pork	Conjunctivitis, and hair loss	Mock and Fontana (2023)
B9	Peanuts, beans, chicken liver, and sunflower seeds	Convulsive seizures and hypertrophy	Hansen and Inselman (2023)
B12	Dairy products, fish, meat, and eggs	Neurological problems, and mouth inflammation	Morales‐Gutierrez et al. (2019)

## 7 ROUTES OF VITAMIN ADMINISTRATION AND ITS IMPACT ON PHARMACOKINETICS

Vitamin A, in the human diet, is primarily available in the form of retinyl palmitate and β‐carotene, which are later enzymatically converted into absorbable retinol in the intestine. However, absorption in the intestine, storage in the liver and associated pharmacokinetics are influenced by genetic makeup in different ethnic groups (Suzuki & Tomita, 2022). Vitamin E depends on dietary fat, bile salts, and pancreatic enzymes for its absorption, where parenteral absorption rates are marginally higher than oral absorption rates (Mohd Zaffarin et al., 2020). Concerning the pharmacokinetic profile of vitamin D, heterogeneity can be observed based on the route of administration, treatment schedules, age, and underlying disease conditions (Fassio et al., 2020). 25‐Hydroxyvitamin D is the most prevalent form of circulating vitamin D, and its presence is not impacted by low‐dose administrations of vitamin D (400–1000 IU/day) (Table 3), making its bioavailability and efficacy dose‐dependent (Chen et al., 2016). Vitamin K, on the other hand, has a scarce presence in systemic circulation due to its rapid metabolism followed by excretion. The inclusion of fats in the diet is reported to increase the absorption of vitamin K3, suggesting a significant role in its pharmacokinetic profile (Mladěnka et al., 2022). It is also interesting to note that injectable formulations of vitamin K are being identified for oral administration to enhance patient compliance (Afanasjeva, 2017).

**TABLE 3:** The different routes of administration explored for the delivery of vitamins.

Vitamins	Routes of administration				Ref.
	Oral	Nasal	Parenteral	Topical	
A	Capsule, Powder		Solution (IM)	Cream, Serum, Patch	Borel and Desmarchelier (2018); Goncalves et al. (2015)
D	Tablet, Capsule, Caplet, Pill, Chewable, Granules, Drops, Solution, Spray	Spray	Emulsion (IM)	Cream	Fassio et al. (2020); Avioli et al. (1967); Chen et al. (2016)
E	Capsule, Granules, Powder		Emulsion (IV, IM, SC)	Hydrogel, Cream, Ointment, Emulsion, Solution, Patch	Eggermont (2006); Brigelius‐Flohé and Traber (1999); Goncalves et al. (2015); Mohd Zaffarin et al. (2020); Julianto et al. (2000)
K	Tablet, Capsule, Pill, Spray, Drops		Solution (IV, IM, SC)	Gel, Cream, Serum	Afanasjeva (2017); Shearer et al. (1970)
B	Tablet, Capsule, Caplet, Pill, Powder, Solution, Drops (Individual and Multivitamin), Elixir (B1)	Spray (B12)	Solution (B1, B6 – IM, IV), Liquid/Solution (B9 – IM, IV, SC), B12 (IM)	Cream (B5), Nail care products (B5), Lotion (B5), Liquid (B7)	Fukuwatari et al. (2009); Zempleni (1995); Allen (2010)
C	Tablet		Infusion (IV)	Lotion, Cream, Spray, Serum	Łukawski et al. (2020); Bansal and Hadimani (2021); Boyer et al. (2005); Carr et al. (2017); Gopi and Balakrishnan (2021)

Vitamin C is primarily absorbed orally; however, in people with habits such as smoking, adequate amounts of vitamin C may be hard to achieve (Lykkesfeldt & Tveden‐Nyborg, 2019). The pharmacokinetics of vitamin C following intravenous infusion show a change from zero order to first order with a constant half‐life independent of the dose administered (Nielsen et al., 2012). The B vitamins have come from plant, yeast, and bacterial origins, and humans must acquire them from sources such as the intestinal microbiota (Yoshii et al., 2019). Therefore, the healthy balance in the gut microenvironment in individuals is one of the primary factors influencing the pharmacokinetics of the vitamin B family. Vitamin B1 has been shown to achieve high systemic levels following oral administration and is absorbed via saturable active transport and non‐saturable passive processes (Table 3) (Smithline et al., 2012). Vitamin B2 is available to the human body via diet and intestinal bacteria. This vitamin uptake is negatively impacted in cases of transport system dysfunction in the small and large intestines (Subramanian et al., 2015). Vitamin B3 can be enzymatically generated in mammals from tryptophan followed by its storage in the liver and is also biosynthesized from tryptophan by intestinal bacteria. Tryptophan metabolism is partially influenced by age, stress, intake of probiotics, and disease conditions, further impacting the pharmacokinetics of vitamin B3 (Gao et al., 2018). Two sources of vitamin B5 (pantothenic acid) include diet and bacteria, wherein the dietary version exits in the form of CoA (coenzyme A), which is further hydrolyzed to the free form in the intestine before absorption. The colonic absorption of the version from bacteria involves colonic carrier‐mediated sodium ion‐dependent transport (Said, 2004b). Impairments in the pathway in the human energy metabolism system are impacted due to hereditary and acquired physiological conditions such as pantothenate kinase‐associated neurodegeneration (PKAN) and medium‐chain acyl‐CoA dehydrogenase deficiency (MCADD). The pharmacokinetics of vitamin B6 reveal that its metabolism is influenced among individuals, which further generates differences in the adverse effects following its consumption in the form of the supplement (Vrolijk et al., 2020). The pharmacokinetic profile of vitamin B6 also differs based on the route of administration, with rapid metabolism occurring in erythrocytes (Zempleni, 1995). Vitamin B7 is absorbed in the small intestine via a sodium ion‐dependent carrier‐mediated transport system, but this is sensitive to the consumption of anticonvulsant molecules (long‐term therapy) such as carbamazepine and primidone (Said et al., 1989). Vitamin B9 (folate) exists in multiple forms, with polyglutamate being a large part of the dietary source, which is hydrolyzed to mono‐glutamate folic acid to aid absorption in the small intestine, after which it is converted to the polyglutamate form in the tissues. The metabolism of the numerous folate forms makes the kinetics complex and further alters the absorption process; for example, pregnancy may alter the time of metabolic equilibrium of the folate supplements (Caudill et al., 1997; Yoshii et al., 2019). Table 3 shows that vitamin B12 was administered via the intramuscular route. However, an alternate intranasal route was studied to compare its pharmacokinetics. It was found that bioavailability following the intranasal route (2%) was comparable to that of the oral route and can be utilized to overcome the pain associated with intramuscular injection (Tillemans et al., 2014).

## 8 DAILY RECOMMENDED DOSE, PRECLINICAL AND CLINICAL FINDINGS OF VITAMINS IN THE DRUG DELIVERY SYSTEM

Vitamins play a crucial role in regulating diverse metabolic and biological functions within the human body. A significant number of individuals worldwide incorporate a daily multivitamin regimen either to address chronic illnesses or as a preventive measure. The widespread dissemination of vitamin‐related information through unregulated advertisements, combined with the easy accessibility of these supplements, has significantly contributed to a high prevalence of their consumption among the general population (Charen & Harbord, 2020). The risk of overdosing primarily depends on whether a vitamin is fat‐ or water‐soluble. Water‐soluble vitamins are absorbed upon digestion and are not stored in tissues for long. In contrast, fat‐soluble vitamins, like those containing iron, can accumulate and be toxic, especially in children (Chang & Rangan, 2011). Excessive vitamin intake leads to consistent symptoms of hypervitaminosis, including headache, fatigue, dizziness, and digestive issues. For fat‐soluble vitamins, the effects are more severe due to tissue accumulation, causing intense intoxication. On the other hand, water‐soluble vitamin toxicity shows milder symptoms and is manageable by increasing urine output and reducing intake (I.J. of H.S. and R. (IJHSR), 2018). To assess the pros and cons of vitamins, a fundamental comprehension of their roles, potential advantages, suggested dosages, and potential interactions with medications or alternative supplements is necessary. For detailed information, please refer to Table 4, which represents the recommended doses of vitamins and the consequences of overdose. Table 5 summarizes the preclinical and clinical data of vitamins.

**TABLE 4:** The recommended dose of vitamins and tolerable limit for patients.

Vitamins	Recommended daily dosage	Upper tolerable limit (UL)	Possible adverse outcomes related to supplement consumption	Ref.
A	Males (>14 years) – 900 mcg Females (>14 years) – 700 mcg Pregnancy (14–18 years) – 750 mcg Pregnancy (19–50 years) – 770 mcg Lactation (14–18 years) – 1200 mcg Lactation (19–50 years) – 1300 mcg	For age (>19 years) – 3000 mcg (about 10,000 IU)	Headache, vomiting, short‐term loss of consciousness, dizziness, irritability, nausea, abdominal pain, fever, skin rashes, and visual disturbances like diplopia and bone pain	Chen et al. (2023); Vitamin and Carotenoids – Health Professional Fact Sheet (n.d.)
C	Males and females (9–13 years) – 25 mg Males (14–18 years) – 75 mg Females (14–18 years) – 65 mg Males (>19 years) – 90 mg Females (>19 years) – 75 mg Pregnancy and lactation – 80 mg and 115 mg	For age (>19 years) – 2000 mg	Gastrointestinal disturbances like diarrhea, nausea, and abdominal cramps	Institute of Medicine (US) Panel on Dietary Antioxidants and Related Compounds (2000)
D	Males and females (31–70 years) – 15 mcg (600 IU) Males and females (>71 years) – 20 mcg (800 IU)	For age (>19 years) – 50 mcg (2000 IU)	Soft‐tissue calcification or hypercalcemia at intake >2000 IU	Wooltorton (2003)
E	Males and females (>19 years) – 15 mg (equals about 22 IU from natural sources of vitamin E and 33 IU from synthetic vitamin E)	1000 mg (nearly 1500 IU natural vitamin E; 2200 IU synthetic)	Nausea, vomiting, diarrhea, possible antiplatelet effects, headache, fatigue and blurred vision at intake >800 IU/d	Wooltorton (2003)
K	Males (>19 years) – 120 mcg Females (>19 years) – 90 mcg	Not known	No reports are available on adverse effects from consumption of excess vitamin K	Institute of Medicine (US) Panel on Micronutrients (2001)
B1	Males and females (9–13 years) – 0.7 mg Males (>14 years) – 1 mg Females (>14 years) – 0.9 mg Pregnancy and lactation (all ages) – 1.2 mg	The evidence on adverse effects is not sufficient to set a Tolerable Upper Intake Level (UL) for Thiamin.	No reports available on adverse effects from the consumption of excess thiamin by ingestion of food and supplements	Institute of Medicine (US) Standing Committee on the Scientific Evaluation of Dietary Reference Intakes and its Panel on Folate, Other B Vitamins, and Choline (1998)
B2	Males and females (9–13 years) – 0.8 mg Males (>14 years) – 1.1 mg Females (>14 years) – 0.9 mg Pregnancy and lactation (all ages) – 1.2 mg	The evidence on adverse effects is not sufficient to set a Tolerable Upper Intake Level (UL) for riboflavin.		Institute of Medicine (US) Standing Committee on the Scientific Evaluation of Dietary Reference Intakes and its Panel on Folate, Other B Vitamins, and Choline (1998)
B3	Males and females (9–13 years) – 9 mg Males (>14 years) – 12 mg Females (>14 years) – 11 mg Pregnancy and lactation (all ages) – 14 mg and 13 mg	For age (>19 years) – 30 mg	There is no indication of negative consequences resulting from the ingestion of niacin naturally found in foods. Nevertheless, certain scientific studies have reported instances of liver damage at doses exceeding 3 grams.	Institute of Medicine (US) Standing Committee on the Scientific Evaluation of Dietary Reference Intakes and its Panel on Folate, Other B Vitamins, and Choline (1998)
B5	Males and females (>19 years) – 5‐7 mg	Not known	Doses greater than 10 g/day may cause mild diarrhea or mild intestinal distress	Sanvictores and Chauhan (2023); Hrubša et al. (2022)
B6	Males and females (9–13 years) – 0.8 mg Males and females (14–50 years) – 1.1 mg Males (>50 years) – 1.4 mg Females (>50 years) – 1.3 mg Females (>14 years) – 11 mg Pregnancy and lactation (all ages) – 1.6 mg and 1.7 mg	For age (>19 years) – 100 mg	Consuming elevated amounts of vitamin B6 through dietary sources has not been associated with any reported negative outcomes. However, chronic administration of 1–6 g oral pyridoxine per day for 12–40 months can cause severe and progressive sensory neuropathy characterized by ataxia	Institute of Medicine (US) Standing Committee on the Scientific Evaluation of Dietary Reference Intakes and its Panel on Folate, Other B Vitamins, and Choline (1998); Vitamin B6 – Health Professional Fact Sheet (n.d.)
B7	Males and females (>19 years) – 30 mcg	Not known	There have been no documented harmful impacts of biotin on humans or animals.	Institute of Medicine (US) Standing Committee on the Scientific Evaluation of Dietary Reference Intakes and its Panel on Folate, Other B Vitamins, and Choline (1998)
B9	Males and females (9–13 years) – 250 mcg Males and females (14–18 years) – 330 mcg Males and females (>30 years) – 320 mcg Pregnancy and lactation (all ages) – 520 and 450 mcg	For age (>19 years) – 1000 mcg	There have been no reported negative outcomes linked to the consumption of typical levels of folate found in enriched foods. However, it has the potential to mask the symptoms of pernicious anemia.	Institute of Medicine (US) Standing Committee on the Scientific Evaluation of Dietary Reference Intakes and its Panel on Folate, Other B Vitamins, and Choline (1998)
B12	Males and females (9–13 years) – 1.5 mcg Males and females (>30 years) – 2 mcg Pregnancy and lactation (all ages) – 2.2 and 2.4 mcg	Insufficient scientific data is available to establish a Tolerable Upper Intake Level (UL) for vitamin B12.	No harmful effects linked to excessive B12 intake from food or supplements in healthy individuals have been identified. Limited evidence from animal studies implies that B12 intake might possibly amplify the carcinogenic effects of certain chemicals.	Institute of Medicine (US) Standing Committee on the Scientific Evaluation of Dietary Reference Intakes and its Panel on Folate, Other B Vitamins, and Choline (1998)

**TABLE 5:** Preclinical and clinical data of vitamins.

Vitamin	Vitamin and types of dosage form	Type of study		Significance in PK	Ref.
		Preclinical	Clinical		
A	Ethosomal hydrogel		20 patients with facial acne vulgaris (18 females and 2 males)	The therapeutic effect of the topical application of ethosomal RP hydrogel on mild to moderate acne. The mean non‐inflammatory lesion number was significantly lower on the RP ethosomal hydrogel formulation treated side.	Salem et al. (2021)
C	Liposomes	CD rats – 60 mg/kg single/dose study carried out to determine the bioavailability of natural vs. synthetic vitamin C	Eleven volunteers in a crossover trial	35% increase in exposure (AUC0–4 h) with a plasma Cmax of about 200 μM after 3 h.	Davis et al. (2016)
D	Nanoemulsion	In vivo study on rats	Clinical studies in children and adult	Relative bioavailability was significantly higher than fat‐soluble D3 by 36% (*p* = .001) based on AUC 0–120. Cmax value was higher by 43% (*p* = .001). In vivo study on rats showed superior intestinal absorption of vitamin D3‐loaded nanostructured lipid carriers	Marwaha and Dabas (2019)
E	PLGA and chitosan nanoparticles	F344 rats	Pharmacokinetics and bioavailability of α‐, γ‐, and δ‐tocotrienols under fed and fasted conditions in eight healthy volunteers.	Nanoparticles containing α‐tocopherol have higher Cmax and AUC0‐∞ values (Cmax: 3.81 and 3.92 μg/mL, respectively; AUC0‐∞: 99 and 80.9 μg/h/mL also successfully improved the bioavailability by 170% and 121%	Simon et al. (2016); Yap et al. (2010)
K	Topical cream application	Wistar rat	–	Vitamin K was beneficial to increase the wound healing rate in animal models by showing an increase in absorption rate.	
B1	Oral delivery of thiamine‐coated nanoparticles	Wistar rats	–	Thiamine‐nanoparticles moved faster from the stomach to the small intestine than naked nanoparticles showing a high rate of absorption	Inchaurraga et al. (2019)
B2	Riboflavin nanoparticles	Immunodeficient mice	–	Vitamin B2 can be photosensitized selectively and efficiently to kill human breast adenocarcinoma cells SK‐BR‐3 and exert a tumouricidal effect with enhancing bioavailability.	Khaydukov et al. (2016)
B3			A crossover randomized clinical trial	Photopic negative response (PhNR) parameters: saturated PhNR amplitude (Vmax) improved by 14.8% [95% CI: 2.8%, 26.9%]	
B5	Tablet dosage form	–	Double‐blind, placebo‐controlled study on 48 subjects	Novel pantothenic acid‐based dietary supplement in subjects with mild to moderate facial acne inflammation reduced with increase absorption of Vit.B5	Yang et al. (2014)
B6	Tablet dosage form	Wistar Rats	Double‐blinded, placebo‐controlled, parallel‐group trial of 90 subjects	Animal studies have reported that inflammation reduces circulating vitamin 6 in rat models.	Ghavidel‐Parsa et al. (2022)
B7	IV Injection	Mice model	–	Synthesized conjugate shows potent anticancer activity in vivo studies with high pharmacokinetics	Rayan et al. (2022)
B9	Solid‐lipid nanoparticle	Mice model		The developed nanoparticles showed higher tumor accumulation and bioavailability	Çetin et al. (2023)
B12	Dry powder inhaler/cyanocobalamin formulated with a proprietary carrier, sodium N‐[8‐(2‐hydroxy benzoyl)amino]caprylate (SNAC)	Wistar Rats	20 healthy male subjects	The oral cyanocobalamin formulation containing SNAC had greater mean absolute bioavailability than the commercial oral formulation (5.09% vs 2.16%, respectively) and reduced Tmax (0.5 h vs 6.83 h, respectively).	Castelli et al. (2011)

## 9 CURRENT CHALLENGES, FUTURE PROSPECTS, AND CONCLUSION

To achieve the desired pharmacological response, it is advisable to examine the pharmacokinetics of the active substance. Primarily, this provides information about the absorption, distribution, metabolism, and excretion of active agents. The use of vitamins to treat deficiencies has been widely publicized worldwide since its inception. Several recent studies have demonstrated their potential applications in the treatment of a wide range of life‐threatening illnesses. To date, various methods for delivering vitamins have been established, including oral, nasal, parenteral, and topical routes. Vitamins can be formulated into various pharmaceutical dosage forms, such as capsules, tablets, pills, granules, drops, and solutions for the oral route. In the case of the nasal route, a nasal spray has been developed for administering vitamin B12. For the parenteral route, solutions, emulsions, infusions, etc., have been developed, while for the topical route, creams, serums, patches, hydrogels, ointments, and other formulations are available. Understanding the pharmacokinetic characteristics of vitamins provides information on their bioavailability, volume of distribution, half‐life, clearance, area under the curve, peak concentration, and peak time. These criteria must be considered during dose formulation for effective management. Despite significant progress in vitamin dosage forms due to their widespread consumption, the majority of vitamin dosage forms are still available in traditional forms. In this context, the relevance of pharmacokinetics in vitamin delivery must be thoroughly explored. In most cases, theoretical models have been used to measure pharmacokinetic parameters, but there is a need to validate these outputs using alternative methods. To achieve the desired therapeutic response, vitamins need to be delivered at the required concentration. Several variables influence vitamin absorption through the oral route, especially vitamin B12 absorption, requiring various dose formulations and proper modes of administration. To optimize pharmacokinetics, modern nanocarriers such as lipid‐based carriers, protein‐based carriers, polymeric carriers, and others must be employed for vitamin delivery. Additionally, tailored delivery to the absorption site alone is necessary to overcome the limitations of oral vitamin administration. Furthermore, more research is needed on the absorption, distribution, and metabolism of vitamin‐based pharmaceutical dosage forms. Preclinical and clinical investigations of advanced dosage forms are essential, and scaling up from lab‐scale to pilot‐scale production is required for novel vitamin dosage forms. It is also crucial to adhere to guidelines provided by regulatory agencies such as the Food and Drug Administration (FDA), the World Health Organization (WHO), and the European Medicines Agency (EMA). Therefore, in vivo experiments and human testing are necessary to ensure biocompatibility, pharmacokinetics, and practical applications. One of the most promising avenues for the future lies in the development and optimization of nanoparticle‐based delivery systems for vitamins. These nanoparticles have the potential to significantly enhance the bioavailability of vitamins by shielding them from degradation, enhancing their solubility, and facilitating controlled release. Researchers have the opportunity to explore various types of nanoparticles, including liposomes, polymeric nanoparticles, and lipid‐based carriers, to fine‐tune the delivery of specific vitamins. Future research could concentrate on customizing vitamin nanoformulations for specific patient populations and health conditions. Tailored formulations could optimize vitamin delivery based on individual pharmacokinetic profiles and dietary requirements, thus maximizing their therapeutic benefits. A deeper comprehension of how vitamins in nanoformulations interact with biological systems and cells is imperative. This includes elucidating the mechanisms of action at the cellular and molecular levels. By studying these mechanisms, researchers can design more effective formulations with targeted therapeutic effects. The prospect of combining vitamins with other therapeutic agents, such as drugs or complementary supplements, presents an exciting avenue for future research. Exploring synergistic effects could enhance the treatment of various diseases, including cancer, cardiovascular disorders, and neurodegenerative conditions. Advancements in pharmacogenomics and personalized medicine may empower healthcare providers to tailor vitamin nanoformulations to an individual's genetic makeup and specific health requirements. This potential development could lead to highly personalized treatments that optimize the efficacy of vitamins while minimizing side effects. The development of biocompatible and environmentally friendly nanoparticles for vitamin delivery should be a central focus for researchers. Sustainable nanocarriers have the potential to reduce the ecological footprint of pharmaceutical production and minimize potential harm to patients. To bring vitamin nanoformulations into mainstream medical practice, extensive clinical trials and regulatory approvals will be indispensable. Collaborations between academia, industry, and regulatory bodies are pivotal to ensuring the safety and efficacy of these innovative formulations. Economically viable production methods for vitamin nanoformulations will be essential to make these therapies accessible to a broader population. Innovative manufacturing processes and economies of scale can help reduce production costs. As these advanced formulations become available, it is crucial to educate healthcare professionals and consumers about their benefits, proper usage, and potential interactions with other medications. This education is paramount to ensuring safe and effective treatment. Finally, the potential global health impact of vitamin nanoformulations should not be underestimated. These formulations, with their improved bioavailability and therapeutic effects, have the potential to play a pivotal role in addressing malnutrition, especially in resource‐constrained regions.

In conclusion, the prospects for vitamin nanoformulations in healthcare are exceptionally promising. As we witness further exploration of the science underpinning these formulations and technological advancements, we can envision a future with a more extensive array of therapeutic applications, enhanced patient outcomes, and a substantial contribution to the overall well‐being of individuals and communities across the globe. In summary, this review article has delved into the realm of vitamin deficiency and underscored the critical role of vitamin pharmacokinetic analysis in enhancing clinical outcomes. It has examined various factors influencing vitamin pharmacokinetics post‐ingestion. The findings presented here illuminate how nanoformulations for vitamin delivery excel in terms of stability, solubility, cellular uptake, and absorption, resulting in superior pharmacological effects for the respective vitamins. Ultimately, this review article has offered a fresh perspective on comprehending vitamin dosage forms and their pharmacokinetics, while also shedding light on current developments and challenges in the field. Looking ahead, the insights provided in this review hold great potential to benefit the scientific community by facilitating the design of advanced and innovative methods for vitamin delivery, ones that offer improved pharmacokinetic performance and, consequently, greater efficacy in clinical applications.

## AUTHOR CONTRIBUTIONS

**Vrashabh V. Sugandhi:** Data curation (equal); formal analysis (equal); investigation (equal); methodology (equal); project administration (equal); resources (equal); software (equal); supervision (equal); validation (equal); visualization (equal); writing – original draft (equal); writing – review and editing (equal). **Rudra Pangeni:** Writing – original draft (equal); writing – review and editing (equal). **Lalitkumar K. Vora:** Resources (equal); software (equal); supervision (equal); validation (equal); visualization (equal); writing – original draft (equal); writing – review and editing (equal). **Sagun Poudel:** Writing – original draft (equal); writing – review and editing (equal). **Sopan Nangare:** Conceptualization (equal); supervision (equal); validation (equal); visualization (equal); writing – original draft (equal); writing – review and editing (equal). **Satveer Jagwani:** Resources (equal); software (equal); validation (equal); visualization (equal); writing – review and editing (equal). **Dnyandev Gadhave:** Resources (equal); software (equal); validation (equal); visualization (equal); writing – review and editing (equal). **Chaolong Qin:** Resources (equal); software (equal); validation (equal); visualization (equal); writing – review and editing (equal). **Anjali Pandya:** Resources (equal); software (equal); validation (equal); visualization (equal); writing – review and editing (equal). **Purav Shah:** Resources (equal); software (equal); validation (equal); visualization (equal); writing – review and editing (equal). **Kiran Jadhav:** Resources (equal); software (equal); validation (equal); visualization (equal); writing – review and editing (equal). **Hitendra S. Mahajan:** Conceptualization (equal); data curation (equal); formal analysis (equal); investigation (equal); methodology (equal); project administration (equal); resources (equal); software (equal); supervision (equal); validation (equal); visualization (equal); writing – original draft (equal); writing – review and editing (equal). **Vandana Patravale:** Methodology (equal); project administration (equal); visualization (equal); writing – review and editing (equal).

## CONFLICT OF INTEREST STATEMENT

All authors declare no conflict of interest.

## CONSENT TO PARTICIPATE

All the co‐authors are willing to participate in this review manuscript.

## CONSENT FOR PUBLICATION

All the authors are willing for the publication of this manuscript.

## ETHICS STATEMENT

This study does not involve any human or animal testing.

## PATIENT CONSENT STATEMENT

This study does not involve any human or animal testing.

## Data Availability

Data sharing not applicable – no new data generated.
